
JOURNAL INFORMATION
==============================
NLM Title Abbreviation: Innov Surg Sci
Journal ID: Innov Surg Sci
NLM Journal ID: 101708165
Journal ID: iss

Innovative Surgical Sciences

EISSN: 2364-7485
Publisher: De Gruyter

ARTICLE INFORMATION
==============================
PMCID: PMC10216884
DOI: 10.1515/iss-2023-9008
Article ID: iss-2023-9008
Article version: 1
Subjects: Article

Abstract DGKCH

Publication date: 2023 Mar
Electronic publication date: 2023 Mar 13
Volume: 8
Issue: Suppl1
First page: 148
Last page: 215
Copyright: © the Author(s), published by De Gruyter
Copyright year: 2023
Copyright holder: the Author(s), published by De Gruyter
License: This work is licensed under the Creative Commons Attribution 4.0 International License.
License URL: https://creativecommons.org/licenses/by/4.0/

Page count: 68

==============================

**Surgery in congenital adrenal hyperplasia in context to §1631e**
ID: 500

**Kategorie: DGKCH - Genitalchirurgie beim nicht einwilligungsfähigen Kind**

Verena Ellerkamp[1], Roland Schweizer[2], Gerhard Binder[2], Tobias Luithle[1], Priska Schneider[3], Regina Müller[4], Jörg Fuchs[1]

[1]*Kinderchirurgie und Kinderurologie, Universitätsklinikum Tübingen, Tübingen, Deutschland*, [2]*UKT Tübingen, Pädiatrische Endokrinologie, Tübingen, Deutschland*, [3]*UKT Tübingen, Abteilung für Psychiatrie, Psychosomatik und Psychotherapie im Kindes- und Jugendalter, Tübingen, Deutschland*, [4]*Universität Bremen, Institut für Philosophie, Bremen, Deutschland*

**Background:**
Since May 2021, an extension of §1631 has been implemented in Germany: gender reassignment surgery in patients with differences of sex development (DSD) before the capacity for self-determined consent needs an interdisciplinary expert opinion and consent of the family court judge. The only exceptional cases are those in which the patient's health would be seriously endangered without such an operation. Congenital adrenal hyperplasia (CAH) is classified to the group of DSD, representing one of the DSD-groups with the highest incidence. Contrary to the current surgical procedure during the first two years of life, CAH girls should therefore grow up with virilized external genitalia until puberty and only then be operated on.

**Materials and methods:**
We report a case of an infant with CAH. The family originates from the Near East and immigrated to Germany only one year before the birth. The case was discussed in the DSD-commission with the aim of preparing an expert opinion for the family court.

**Results:**
At birth the child was initially assigned and named as male. After diagnosis of CAH with external genitalia Prader score V the family insisted on gender reassignment surgery. No urinary tract infections occurred, micturition was normal, endocrinological treatment was well tolerated without relevant reduction of the enlarged clitoris. Therefore, there were no medical reasons for early surgery, and initially, the commission failed to find agreement on recommendation for surgery. Finally, after intensive interdisciplinary follow-up of the parents and the child by psychologists, ethicists, social paediatricians, endocrinologists, and paediatric surgeons, a consensus decision was reached pro surgery. This was mainly based on the fact, that the psychological assessment showed that the family would reject the child due to virilization. The family court judge's approval was thereafter given quickly. Surgery was performed at the age of 23 months in form of a clitoral reduction and feminizing

Open Access. © 2023 The Author(s), published by De Gruyter. This work is licensed under the Creative Commons Attribution 4.0 International License.

genitoplasty without vaginoplasty. The correction of the urogenital sinus is planned after onset of menarche.

**Conclusion:**

The new legislation on gender reassignment complicates the treatment of CAH girls. One may assume, growing up with servere virlized external genitalia in girls would cause severe psychological problems in these patients. However, there are currently no studies on this topic. Close interdisciplinary cooperation and the development of special psychological expertise are necessary to ensure adequate treatment of these patients also in the future.,

**hormone receptors in hypospadias & controls**

ID: 786

**Kategorie: DGKCH - Genitalchirurgie beim nicht einwilligungsfähigen Kind**

Michael Sennert[1,2,3], Christina Perske[4], Johannes Wirmer[1], Mohamed Fawzy[1], Ahmed Hadidi[1]

[1]*Sana Klinikum Offenbach GmbH, Offenbach am Main, Deutschland*, [2]*Universitätsklinikum Hamburg-Eppendorf, Hamburg, Deutschland*, [3]*Altonaer Kinderkrankenhaus, Hamburg, Deutschland*, [4]*Institut für Pathologie, Göttingen, Deutschland*

**Background:**

To study hormone receptor expression in children with hypospadias and compare them to controls.

**Materials and methods:**

Foreskin samples were collected from 35 children during hypospadias operations (18 distal and 17 proximal hypospadias) and compared with foreskin samples of a control group of 33 children (15 age matched and 18 older boys) during circumcision.

The samples were stained with H/E, androgen (AR), estrogen (ER) and progesterone receptors (PR). The receptors were blindly evaluated. An Allred score was used to evaluate receptor expression in both the epithelium as well as stroma.

**Results:**

AR were also detected in all cases. AR expression in the stroma was more evident than in the epithelium.

AR expression in the hypospadias groups was less than the age matched controls ($p<0,05$). There was no significant difference in between the two hypospadias groups nor between the two control groups.

Older control group showed significantly elevated levels of AR expression compared to hypospadias group and younger control group ($p<0,05$).

ER were also detected all cases (except in one control with lichen sclerosis). The stroma showed more ER than in epithelium.

PR was minimal or negative in all samples.

**Conclusion:**

Boys with hypospadias showed significantly weaker expression of Androgen Receptors than age matched controls. The severity of Hypospadias did not influence hormone receptor distribution. AR expression is better observed in the stroma than in the epithelium.

There was no difference in ER expression between the hypospadias group (distal or proximal) and age matched normal controls.

**"Analysis of German insurance claims indicate high rate of reoperations after hypospadias surgery"**
ID: 917

**Kategorie: DGKCH - Genitalchirurgie beim nicht einwilligungsfähigen Kind**

Alejandro Hofmann[1], Torben Schmidt[2], Jochen Blaser[3], Jan Zeidler[2], Benno Ure[1], Joachim Kübler[1]

[1]*Hannover Medical School, Department of Pediatric Surgery, Hannover, Deutschland*, [2]*Leibniz University, Center for Health Economics Research Hannover , Hannover, Deutschland*, [3]*Techniker Health Insurance, Hannover, Deutschland*

**Background:**
Corrective surgery of hypospadias is recommended to be performed within the first years of life to avoid surgical and psychological complications. Nonetheless, the indication for surgery in distal forms without relevant penile curvature is somewhat controversial. Although there are numerous studies regarding different techniques, reliable data regarding outcome is scarce. We used a German health insurance claims database covering a population of approximately 9 million to investigate the rate of early reoperations after hypospadias surgery.

**Materials and methods:**
Data were extracted from the largest German statutory health insurance TK (~9 million clients) in a 4.5-year period (2018-2022). All patients aged 0-17 years with ICD-10 code "hypospadias (Q54.0-Q54.3)" as well as designated OPS-Codes were included.

**Results:**
A total of 1570 Patients were included in the study, including 703 glandular (Q54.0), 691 penile (Q54.1), 72 penoscrotal (Q54.2) and 48 perineal hypospadias. During the study period, 1230 patients received only one operation. 340 patients received more than one operation, in total 430 reoperations. Of these, 59 were planned staged procedures. Other reoperations were either corrections of strictures or fistulas (n=127) or complete reconstructions after failed prior reconstruction (n=244), for a total rate of 24% unplanned reoperations. Unplanned reoperation rates were less in glandular forms (~11%) compared to penile (~25%) and more proximal forms (>50%).

**Conclusion:**
Insurance claims data indicate a high rate of reoperations after hypospadias surgery within few years postop. As only a limited observational period was included in this study the factual rate of reoperations is likely to be higher. A systematic assessment of short and long-term complications as well as cosmetic and psychological benefits after hypospadias surgery is warranted

### Determination of Tissue Transit Time (TTT) of 99mTc-mercaptoacetyltriglycine (99mTc-MAG3) diuretic renography in infants with ureteropelvic junction obstruction-a multicenter prospective observational study-

ID: 48

**Kategorie: DGKCH - Herausforderung Früh- und Neugeborenenchirurgie**

Tobias Luithle[1]

[1]*Univerity Children`s Hospital Tübingen, Department of Pediatric Surgery and Pediatric Urology, Tübingen, Deutschland*

**Background:**

To evaluate the prognostic value of $^{99m}$Tc-MAG3 tissue transit time (TTT) for the development of differential renal function (DRF) in infants with unilateral ureteropelvic junction obstruction (UPJO) in a prospective observational multicenter study.

**Materials and methods:**

Infants below 3 months of age with a unilateral isolated hydronephrosis ≥ grade 3 received ultrasound (US) and $^{99m}$Tc-MAG3 diuretic renography (DR) at two different time points (Investigation1 and Investigation2). Data were analyzed at local centers and at the study center. TTT was determined for each DR, inter-observer variation for TTT and standard parameters for judgement of DRF development were assessed.

**Results:**

Data of 37 patients were analyzed. Median age of the children was 11 weeks (7-15) at Investigation1 and 26 weeks (19-33) at Investigation2. A delayed TTT at Investigation1 was not associated with deterioration of DRF at Investigation2 in both, locally (10/37 cases) and centrally (4/37) analyzed cases. However, sensitivity and specificity were poor. The intraclass correlation coefficient (ICC) comparing local and central findings of TTT and diuretic curve patterns demonstrated poor or fair agreement. Analysis of standard parameters for DRF development revealed a prognostic value only for the dichotomized anteroposterior renal pelvic diameter (APD, p=0.03, 95%-CI 1.2-22.2).

**Conclusion:**

We present the first prospective multicenter analysis of TTT to determine DRF development in infants with UPJO. In the presented setting TTT was not useful as marker for this purpose. Sensitivity and specificity of TTT were not sufficient for risk stratification. Improved utility of TTT as marker might be achieved using a different study setting.

**Laparoscopic Suture-Lasso Inguinal Hernia Repair in the Newborn - A Simulator for Pediatric Surgery Trainees**

ID: 408

**Kategorie: DGKCH - Herausforderung Früh- und Neugeborenenchirurgie**

Julia Haehl[1], Andreas Lindner[1], Oliver Muensterer[1]

[1]*Kinderchirurgische Klinik und Poliklinik im Dr. von Haunerschen Kinderspital, München, Deutschland*

**Background:**

Inguinal hernias are among the most common pediatric conditions requiring surgery. Not only the overall incidence, but also the incidence of bilateral inguinal hernias is significantly higher in premature newborns compared to term newborns. Recently, minimally invasive pediatric techniques have had widespread acceptance for hernia repair. In comparison with open surgery, the laparoscopic approach offers the opportunity to explore and repair the contralateral side to prevent metachronous hernia and the repair of bilateral hernias is quicker than via open technique. However, there is a relevant learning curve for laparoscopic hernia repair, especially when dealing with premature infants. The learning curve for complex surgery in pediatric surgery has gained relevance due to resident duty hour restrictions and due to increased safety awareness in the operating room. We therefore developed a laparoscopic hernia repair training model for pediatric surgery trainees in our department, to flatten their learning curve before performing laparoscopic hernia repair on patients.

**Materials and methods:**

A complex task trainer was developed to simulate laparoscopic hernia repair in the male and female newborn. Low budget materials such as molded silicone, three dimensionally printed plastic and everyday objects such as condoms to imitate the hernia sack were used. A study to evaluate face, content and construct validity is planned using two cohorts of pediatric surgeons (trainees and experts). We plan to recruit 30 subjects in each group, performing simulated hernia repair using the model 3 times and then compare completion times. We also plan to survey the subjective feel, realisms, and usefulness of the model among participants.

**Results:**

The simulator consists of a molded silicone model to imitate the abdomen of the newborn baby. The inguinal canal is created by a condom, which is pulled from the outside to the inside of the model. The opening of the condom thus comes to lie inside the silicone model and is folded over a plastic ring so that the latex comes to lie circularly on the silicone. In the center of the latex ring, the condom is pulled outward through the silicone, thereby simulating the open processus vaginalis peritonei. The left side of the model simulates the male anatomy with a yellow loop as the ductus deferens and a blue loop as testicular vessels (Fig. 1). The right side of the model simulates the female anatomy with a bigger loop as the ligamentum rotundum (Fig. 2).

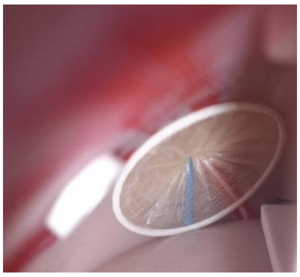

**Fig. 1**

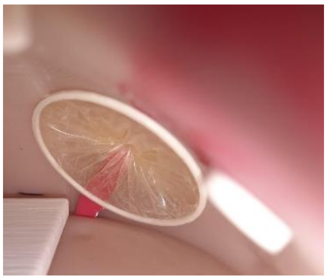

**Fig. 2**

The operation itself comprises of a suture lasso technique with two Prolene 4-0 loops, which are placed through the silicone and around the inguinal ring with a Tuohy needle from the medial and lateral sides. Hereby the percutaneous needle-dissection of the peritoneum and the suture lasso technique is simulated. The model was completely built from low-cost material (in total under 20 Euro) and was easily reproducible due to a 3d printed mold. It was therefore integrated into the training program “SuSiPed” for trainees in our department in the form of the above-mentioned study.

**Conclusion:**

A simulation model for training laparoscopic herniotomy in pediatric surgery is shown. Validity data will be available after completion of the above-mentioned study.

**Postnatal surgical complications in lower urinary tract obstruction following fetal vesico-amniotic shunting**
ID: 560

**Kategorie: DGKCH - Herausforderung Früh- und Neugeborenenchirurgie**

Anna Meßling[1], Matthias Nissen[2], Jochen Hubertus[2], Ahmed Natsha[2], Volker Sander[2], Christoph Berg[3]

[1]*Ruhr- Universität Bochum, Klinik für Kinderchirurgie, Marienhospital Witten, DE, Witten, Deutschland*,
[1]*Ruhr- Universität Bochum, Klinik für Kinderchirurgie, Marienhospital Witten, DE, Witten, Deutschland*,
[3]*Klinik für Pränatalmedizin, Marienhospital Witten, Witten, Deutschland*

**Purpose:**
Fetal lower urinary tract obstruction (LUTO) forms part of the congenital anomalies of kidney and urinary tract spectrum, which is among the most frequent causes for end stage kidney disease in children. Posterior urethral valves, followed by urethral atresia and prune belly syndrome are the most common causes for LUTO. The associated oligohydramnion may lead to pulmonary hypertension and explains the high mortality rate in these children. Fetal management includes early vesicoamniotic shunting (VAS), a technique that has been evolved over the last years to preserve kidney function. However, VAS is associated with postnatal complications such as shunt dislocation or iatrogenic gastroschisis. Herein, we report our initial experiences with two cases that required postnatal surgical intervention related to fetal VAS and obstructive uropathy.

**Materials and Methods:**
We describe our preliminary experience with the postnatal management of two neonates following intrauterine VAS for LUTO treated at our tertiary pediatric surgery hospital between 2021 and 2022. The data was retrospectively analyzed in relation to perioperative characteristics focusing on complication spectrum, type of intervention, and the clinical outcome on follow-up assessment. For VAS, the Somatex® shunt, as described by Strizek et al. in 2020, was utilized.

**Results:**
Five cases of LUTO were identified being treated at our hospital during study period. Two patients (40%) underwent intrauterine VAS within first trimester and each required postnatal surgical intervention. Weight at birth was >3kg and gestational age was >37 weeks in both cases. VAS dislocation occurred two times in each patient, requiring an additional stenting.
In one patient, the vesicoabdominal stent was removed after birth and a bladder perforation was sutured by laparoscopy. In the second patient, dislocated abdomino-amnial stents were removed at subcutaneous level and laparotomy was performed to treat an incarcerated omentum prolapse. In both cases, a suprapubic catheter secured postnatal urinary drainage in the initial surgery.

In the first patient, underlying diagnosis was urethral atresia in forms of a congenital urethral stenosis near to the bladder neck. In the second patient, posterior urethral valves were identified.
On follow up the two boys did not suffer from renal insufficiency with creatinine and cystatine c levels within normal age-adjusted values.

**Conclusion**:
In LUTO patients treated with fetal VAS, stent dislocation is a frequent complication. While an early surgical management of LUTO associated complications can be necessary, obtained complications secondary to stent dislocation and its treatment were of minor extent.

**Reduction of neonatal depressed skull fractures using vacuum-therapy**
ID: 579

**Kategorie: DGKCH - Herausforderung Früh- und Neugeborenenchirurgie**

Ahmed Natsha[1], Volker Sander[2], Anna Messling[3], Ralf-Bodo Tröbs[4], Jochen Hubertus[2], Matthias Nissen[2]

[1]*kinderchirurgie , Witten, Deutschland*, [2]*kinderchirurgie, Witten, Deutschland*, [3]*kinderchirurgie, witten, Deutschland*, [4]*kinderchirurgie, duisburg, Deutschland*

**Background:**
Depressed skull fractures often occur after traumatic birth. Current treatment of these age-specific fractures is controversial. While some authors refer to the possibility of a spontaneous resolution, others suggest a surgical elevation maneuver. Recently, a non-surgical, vacuum-induced fracture reposition has been proposed in small case series. We present our preliminary experience in two neonates in which depressed skull fracture was successfully treated by vacuum application.

**Materials and methods:**
In two neonates, isolated skull fracture was confirmed radiologically without further intracranial pathologies. A pediatric resuscitator mask connected to a 50ml syringe was utilized.

**Results:**
A seven-days-old female (case 1) and a two-days-old male neonate (case 2) presented with a depressed fracture of the right parietooccipital skull following vacuum extraction during vaginal delivery under cephalic presentation (case 1) and cesarean section after prolonged and obstructed labor under cephalic presentation (case 2). Postnatal adaptation and neurological status were inconspicuous in either case. Both patients underwent successful vacuum-induced reposition of fracture under procedural anesthesia. Further course was unremarkable in either case.

**Conclusion:**
Birth traumatic skull fractures are rare[3], and minor defects typically resolve spontaneously[2]. In cases of isolated skull fracture and depression depth > 5mm without associated injuries, vacuum reposition constitutes a considerable treatment option, preferably performed within first 72 hours prior to start of fracture remodeling[1,3]. Sealing of resuscitator mask in case 2 was established by application of Instillagel© (Farco-Pharma, Cologne, Germany) lubricant in order to avoid the commonly proposed method[1,3] of shaving the newborns head, as performed in case 1. Herein applied minor modification of technique by lubricant application for vacuum sealing in lieu of local head shaving has not been previously reported and might also contribute to parental contentedness. In conclusion, selected cases of simple neonatal depressed skull fracture might be amenable to the minimal invasive and affordable vacuum-induced reposition maneuver.

### Congenital pulmonary malformations: Outcomes after thoracoscopic and open surgical resection in infancy

ID: 661

**Kategorie: DGKCH - Herausforderung Früh- und Neugeborenenchirurgie**

Benjamin Mayer[1], Leon Ole Schöneberg[1], Frank Fideler[2], Jürgen Schäfer[3], Felix Neunhoeffer[4], Hans Joachim Kirschner[1], Justus Lieber[1], Jörg Fuchs[1]

[1]*Universitätsklinikum Tübingen - Klinik für Kinderheilkunde und Jugendmedizin, Kinderchirurgie und Kinderurologie, Tübingen, Deutschland*, [2]*Universitätsklinik für Anästhesiologie und Intensivmedizin, Kinderanästhesie, Tübingen, Deutschland*, [3]*Radiologische Universitätsklinik Tübingen, Kinderradiologie, Tübingen, Deutschland*, [4]*Universitätsklinikum Tübingen - Klinik für Kinderheilkunde und Jugendmedizin, Kinderkardiologie, Intensivmedizin und Pulmologie, Tübingen, Deutschland*

**Background:**

Congenital pulmonary malformations are rare but are increasingly diagnosed during prenatal ultrasound examinations. After birth, clinical symptoms range from asymptomatic to life-threatening conditions. Asymptomatic patients are at relevant risk for pulmonary infections, air leaks, and malignant transformation later in life. While the indication for surgical treatment in respiratory unstable patients is clear, the indication and timing of surgical treatment in asymptomatic patients remains controversial. The aim of this work was to analyze the timing, technique, and outcome of surgical treatment in children with congenital pulmonary malformations.

**Materials and Methods:**

All patients with congenital pulmonary malformations treated at our institution between 2002 and 2019 were retrospectively evaluated. Patients diagnosed prenatally with congenital pulmonary malformation underwent prenatal imaging by serial ultrasonography and fetal magnetic resonance imaging if treated during the later study period. Preoperative diagnostic imaging included feed-and-sleep magnetic resonance imaging or contrast-enhanced angio-computed tomography using a dedicated low dose pediatric protocol. Surgery was performed in asymptomatic patients between 3 and 6 months of age to reduce perioperative complications and duration of mechanical ventilation. The type and timing of surgery were determined based on respiratory status and previous infections. Patients with thoracoscopic resection of congenital pulmonary malformations were compared with patients in whom resection was performed by thoracotomy.

**Results:**

Between 2002 and 2019, 76 patients underwent surgery for congenital pulmonary malformations at our institution. After histopathologic examination congenital pulmonary airway malformation was diagnosed in 37 patients, bronchopulmonary sequestration in 13 patients, bronchogenic cyst in 9

patients, congenital lobar emphysema in 8 patients, hybrid lesion and middle lobe syndrome in 3 patients each, pulmonary cyst in 2 patients, and an accessory lobe in 1 patient. Surgery was performed in all patients before they were 6 months old. Urgent surgery for respiratory failure via thoracotomy was required in 10 newborn patients. Resection of a congenital pulmonary malformation was performed thoracoscopically in 53 patients (mean age 5.2 months, mean weight 9.5 kg, mean operative time 181.4 minutes) and by thoracotomy in 23 patients (mean age 1.2 months, mean weight 6.9 kg, mean operative time 142.3 minutes). The conversion rate for thoracoscopic surgery was 14.5% and decreased to 8.7% during the last 5 years of the study period. Patients who underwent thoracoscopic surgery recovered faster postoperatively than patients who underwent thoracotomy with shorter duration of ventilation (mean 0.5 vs. 2.3 days; $p < 0.001$), shorter intensive care stay (mean 1.6 vs. 5.2 days; $p < 0.001$), and shorter hospital stay (mean 7.9 vs. 12.9 days; $p = 0.02$). No complications greater than grade III of the Clavien-Dindo classification were observed in either group. At regular follow-up (mean 34 months), 49 of the 53 patients who underwent thoracoscopic surgery showed no complaints. After thoracotomy, rib fusions were observed in 5 patients and thoracic wall asymmetries in 4 of 23 patients.

**Conclusion:**

State of the art treatment of congenital pulmonary malformations begins prenatally and includes an interdisciplinary coordinated treatment plan. In respiratory unstable patients resection should be performed via thoracotomy. In asymptomatic patients, elective thoracoscopic resection in early infancy can be performed safely with good outcome. Therefore, thoracoscopic resection of congenital pulmonary malformations should be offered to patients in centers with appropriate expertise as an alternative to thoracotomy.

**Implementing the German registry for congenital malformations KiRaFe**

ID: 799

**Kategorie: DGKCH - Herausforderung Früh- und Neugeborenenchirurgie**

Andrea Schmedding[1], Udo Rolle[1], Jessica Vasseur[2], Jens Göbel[2], Holger Storf[2]

[1]*Frankfurt am Main, Goethe-Universität, Universitätsklinik, Klinik für Kinderchirurgie und Kinderurologie, Frankfurt am Main, Deutschland*, [2]*Frankfurt am Main, Goethe-Universität, Universitätsklinik, Institut für Medizininformatik, Frankfurt am Main, Deutschland*

**Background:**

Congenital malformations of the abdomen and spine are rare disorders that usually require neonatal surgery and will be associated with lifelong health implications. Patient care for congenital malformations is decentralized in Germany, making it difficult to obtain large structured datasets, which would help to improve the management and quality of life of affected children.

Therefore, KiRaFe (KinderRegister für angeborene Fehlbildungen), a nationwide registry for congenital malformations, was initiated by the German Society of Pediatric Surgery with the primary goal of measuring the quality of care for selected malformations, including patients with multiple malformations.

**Materials and methods:**

KiRaFe is based on the Open Source Registry System for Rare Diseases (OSSE), a software framework to build web-based patient registries. The parameters to be included in the registry were initially discussed in expert groups, taking into account compatibility with (inter-)national registries, and documented in Excel sheets as templates for implementation in OSSE.

**Results:**

The development of the registry started in 2015. In 2021, the first patients could be enrolled in the registry; eleven months onwards 14 different departments have registered 171 patients.

The main challenge was and is the fact that children can have more than one congenital malformation to be documented. Since the time required for documentation is a crucial factor for the acceptance of a registry, steps had to be taken to modularize the documentation. The resulting modular set of >500 parameters includes general and disease-specific contents for eight malformations, allowing future extensions for additional conditions or specialties. Depending on the selected diagnosis and context, only the relevant forms are visible to the users, corresponding to the registry's modular design.

Data protection requirements also necessitated several technical adjustments to the OSSE software. No actual dates are stored in the registry to prevent a patient's date of birth from being deduced from a given date. Instead, the patient's day of life is automatically calculated and episodes can be linked to index values rather than exact dates.

**Conclusion:**
We successfully launched the nationwide KiRaFe registry for congenital malformations which will facilitate systematic research into the incidence, treatment, and clinical course of these conditions. Due to its modular design, KiRaFe has the potential to be expanded to include additional malformations, specialists, and centers in the future.

**Acknowledgments**
We would like to thank the “Dr. Emil Alexander Huebner und Gemahlin-Stiftung” for their financial support of the registry.

**Intrauterine volvulus – Outcome and management of a prenatal emergency**
ID: 904

**Kategorie: DGKCH - Herausforderung Früh- und Neugeborenenchirurgie**

Maximilian Holweg[1], Karl Kagan[2], Andreas Schmidt[1], Justus Lieber[1], Jörg Fuchs[1]

[1]*University Hospital Tübingen, Department of Pediatric Surgery and Pediatric Urology, Tübingen, Deutschland*, [2]*University Hospital Tübingen, Department of Obstetrics and Gynecology, Tübingen, Deutschland*

**Background:**
Midgut volvulus is an acute and severe disease with a serious morbidity and mortality. Delay of diagnostic and therapy is associated with ischemia and necrosis of bowel and leads to mortality and short bowel syndrome with (lifelong) dependence on parenteral nutrition. Most midgut volvuli occur within the first year of life. Therapy is emergency surgery with derotation of the volvulus and correction of intestinal abnormal rotation, if present. Intrauterine volvulus is a rare prenatal emergency, diagnostic is challenging, and emergency caesarean section and immediate surgery of the newborn is necessary. The aim of this work was to analyse the management and outcome in children with intrauterine volvulus.

**Materials and methods:**
All patients with midgut volvulus treated at our institution between 2005 and 2022 were retrospectively evaluated, including prenatal suspected volvulus. Surgery was performed immediately after diagnosis. Outcome, morbidity and mortality after surgery were evaluated.

**Results:**
Between 2005 and 2022, 53 patients with volvulus underwent emergency surgery in our institution. 10 of those patients had prior abdominal surgery and will not be considered further. Of the remaining 43 patients, 37 (86 %) underwent surgery within the first year of life and 23 (53,5 %) showed malrotation. 24 patients were mature born and 19 were premature babies. The premature babies had an average of 29,2 completed weeks of gestation. 5 patients died after surgery; all were extreme premature babies with an average of 25 completed weeks of gestation. 3 patients suffered from short bowel syndrome and long-term parenteral nutrition, one of them was successfully weaned from parenteral nutrition, another patient is currently in the weaning process. 5 patients (11,6 %) were diagnosed with intrauterine volvulus by prenatal ultrasound and immediate caesarean section was performed. All patients were premature babies with an average age of 32,8 completed weeks of gestation and an average weight of 2148 grams. The patients underwent immediate surgery after birth. Volvulus was derotated, all 5 patients had a normal bowel anatomy and in 4 patients resection of a part of the small bowel and ileostomy was performed. In 1 patient the volvulus was just derotated. All patients with intrauterine volvulus received oral food. The patient without ileostomy was readmitted to hospital with a severe necrotising enterocolitis, half of the small bowel had to be removed via laparotomy and ileostomy was performed. This patient suffered from short bowel syndrome and needed long term parenteral nutrition but is in the weaning process from parenteral nutrition now and receives partial enteral nutrition. In all 5 patients, ileostomy was closed in the following months. No patient with intrauterine volvulus died. Just 3 of 43 patients (7 %) with volvulus were later diagnosed with cystic fibrosis, but looking at the intrauterine volvulus group, 2 of 5 patients (40 %) suffered from cystic fibrosis.

**Conclusion:**
State of the art treatment of volvulus is emergency surgery and derotation of the volvulus. Short ischaemia time is essential to prevent irreversible damage and short bowel syndrome. In patients with intrauterine volvulus, emergency caesarean section and immediate surgery of the newborn should be performed if the fetus is developed enough to survive the procedure. There seems to be a correlation between intrauterine volvulus and cystic fibrosis and further diagnostic is advised.

**Tracheal aspirate-derived basal stem cells provide a viable model to study epithelial defects in patients with congenital diaphragmatic hernia**

ID: 56

**Kategorie: DGKCH - Kinderchirurgische Forschung**

Richard Wagner[1,2], Gaurang Amonkar[2,3], Wei Wang[2], Kamakshi Bankoti[2], Jessica Shui[2], Andrew Tse[4], Richard Keijzer[4], Frances High[2], Patricia K. Donahoe[2], Paul H. Lerou[2], Xingbin Ai[2]

[1]*Uniklinikum Leipzig , Klinik für Kinderchirurgie*, [2]*Massachusetts General Hospital, Boston, United States*, [3]*Massachusetts General Hospital, Department of Neonatology*, [4]*University of Manitoba, Bannatyne Campus, Winnipeg, Canada*

**Background:**

Lung hypoplasia is the leading cause of death in CDH patients, but the pathoetiology remains poorly understood. To overcome difficulties of accessing neonatal lung tissue, we developed a robust method to derive airway basal stem cells (BSCs) from tracheal aspirates. CDH patient-specific BSCs provide an *in vitro* model for basic research of epithelial defects in CDH.

**Materials and methods:**

Tracheal aspirates were obtained from term (>37 weeks) CDH newborns (n=12) and term controls (n=11). $P63^+Keratin5^+Nkx2.1^+$ BSCs were isolated and cultured for 2-3 weeks prior to bulk RNA sequencing and Air-Liquid-Interface (ALI) differentiation. Pathology specimens from fetal CDH lungs (n=4) and E21 rat lungs (nitrofen (n=4), nitrofen + hernia (n=4) and controls (n=4)) were immunostained.

**Results:**

We established 12 CDH BSC lines. Compared to full-term control BSCs, CDH ALI-cultures display defective differentiation, manifested by greater retention of basal cell marker Keratin 5 and a reduction in secretory lineages. We confirmed increased epithelial Keratin 5 expression in human fetopsy CDH lungs and in nitrofen CDH tracheas. Transcriptomic assays revealed > 2000 differentially expressed genes between CDH and control BSCs that are enriched in inflammatory and epithelium-to-mesenchyme transition pathways. Finally, we show that dexamethasone treatment can completely rescue the CDH differentiation phenotype.

**Conclusion:**

Taken together, BSCs derived from TA samples of CDH newborns partially reproduce the differentiation defect of BSCs *in vivo*. Therefore, these cells provide a surrogate to epithelial progenitors in CDH patients to study the pathogenesis of lung defects and for *in vitro* testing of potential novel therapeutics.

### ACTIVATED NFkB AND ITS DOWNSTREAM TARGETS ARE INCREASED IN NITROFEN INDUCED LUNG HYPOPLASIA AND CAN BE RESTORED VIA DEXAMETHASONE OR NFKB INHIBITOR TREATMENT

ID: 57

**Kategorie: DGKCH - Kinderchirurgische Forschung**

Florentine L. Dylong[1], Jan Riedel[1], Nicole Peukert[1], Paula Lieckfeldt[1], Xingbin Ai[2], Martin Lacher[1], Jan Hendrik Gosemann[1], Richard Wagner[1]

[1]*Universitätsklinikum Leipzig, Klinik und Poliklinik für Kinderchirurgie, Leipzig, Deutschland*, [2]*Massachusetts General Hospital, Boston, United States*

**Background:**

Lung hypoplasia in CDH (congenital diaphragmatic hernia) is a common developmental defect with significant neonatal mortality but unclear pathophysiology. We recently proposed, that inflammatory processes are enriched during abnormal lung development in CDH. Here, we studied the proinflammatory NFKB (p65) pathway in nitrofen induced lung hypoplasia.

**Materials and methods:**

We used an *ex vivo* nitrofen-model to mimic lung hypoplasia without mechanical compression of the lung and the established nitrofen CDH model in vivo (E9). Fetal E14.5 lung explants were exposed to DMSO (control, n=42), nitrofen (n=48), nitrofen + dexamethasone (n=32) or nitrofen + NFKB inhibitor (n=25) and cultured for 1-3 days. We monitored lung growth and budding daily and measured activated p65 protein (pp65; S536) and mRNA abundance of NFkB target genes (e.g.: TNF-α, CXCL1, Myc). Dexamethasone was also applied intraperitoneally to nitrofen treated pregnant dams at E10, E11 and E12.

**Results:**

We observed significantly reduced lung budding and lung growth in nitrofen-exposed hypoplastic lung explants. The NFkB activated subunit p65 (pp65) was significantly increased in nitrofen lungs after 24h and 48h in culture and *in vivo* nitrofen CDH lungs. NFkB downstream targets *TNF-α, CXCL1, TLR4* and *MYC* showed increased mRNA levels in nitrofen exposed lungs, and TNF-α protein was significantly increased in the airway epithelium of nitrofen CDH lungs at E21. Human bronchial epithelial cells also enhanced their NFkB activation after nitrofen exposure in culture. *Ex vivo* dexamethasone or specific NFkB inhibitor treatment restored abnormal lung branching and normalized NFkB and TNFa signaling ex vivo. Lastly, dexamethasone in vivo treatment increased canalicular lung budding in nitrofen exposed pups.

**Conclusion:**

Nitrofen exposed lungs show increased NFkB p65 activation associated with induction of the proinflammatory downstream target TNF-α. Dexamethasone and specific NFkB inhibitor treatment can rescue the abnormal lung branching and normalize activated NFkB *ex vivo* and *in vivo*.

**Biliatresone – an Isoflavonoid leading to clinical signs of biliary atresia in murine neonates.**
ID: 109

**Kategorie: DGKCH - Kinderchirurgische Forschung**

Hans Christian Schmidt[1], Johanna Hagens[1], Pauline Schuppert[1], Laia Pagerols Raluy[1], Magdalena Trochimiuk[1], Birgit Appl[1], Konrad Reinshagen[1], Christian Tomuschat[1]

[1]*University Medical Center Hamburg-Eppendorf, Hamburg, Germany, Department of Pediatric Surgery, Hamburg, Deutschland*

**Background:**
Biliary atresia (BA) affects the intra- and extrahepatic bile duct system and is characterized by inflammation, fibrosis, and obliteration. The lack of knowledge regarding the pathophysiology has restricted the development of new treatment [1-3]. Only the Kasai surgery and liver transplantation are available as treatments [4]. It is important to highlight the regional variance in BA incidence, which is higher in Asia and the Pacific [5]. In Taiwan and French Polynesia, the incidence varies between one in 3500 and 10,000, compared to one in 15000 to 25000 in Europe, England, Wales, and Canada [6-15]. Studies looking at immunological, viral, genetic, and vascular etiologies were unable to clarify the pathophysiology and largely produced inconsistent findings [8, 16-19]. There is currently no animal model that enables a precise simulation of BA in all of its clinical variability [20]. It is known that the Isoflavonoid biliatresone causes the transcription factor SOX17 to be downregulated and Glutathione (GSH) to be reduced [21, 22]. In our experiments, we used C57BL/6J-neonates to assess the hypothesis that biliatresone induces BA in murine neonates [23]. We demonstrated that biliatresone is an effective inductor for cholangiopathies.

**Materials and methods:**
Following ethical approval (N045/21), Biliatresone was injected intraperitoneally in C57BL/6J-neonates within the first 36 to 48 hours of life. A saline-based solution containing the same amount DMSO as the treatment solution served as a control. Neonates were euthanized seven days after injection followed by blood collection, harvesting of the liver and the extrahepatic biliary system as described previously [24]. Serum samples were analyzed for gamma-GT, alkaline phosphatase, albumin, bilirubin, ALT und GLDH. Tissue samples were embedded in paraffin, cut in 2µm-slides and processed for staining.

**Results:**
The injection of Biliatresone in C57BL/6J-neonates resulted in clinical signs of BA, which occurred no earlier than two days after the injection. Treated animals developed jaundice, ascites, acholic stools, yellow urine and delayed weight gain. The gallbladders of neonates with persistent jaundice were hydropic and extrahepatic bile ducts were twisted and dilatated. However, recovery from jaundice and only slightly impaired development without jaundice despite toxic treatment has also been observed.

**Conclusion:**

Our clinical findings suggest that Biliatresone is a promising tool for inducing cholangiopathies in C57BL/6J-neonates, such as biliary atresia. Taking previous data into consideration [23] we assume an absence of lumen and fibroblastic proliferation of the extrahepatic biliary system and intrahepatic mild fibrosis surrounding the periportal region.

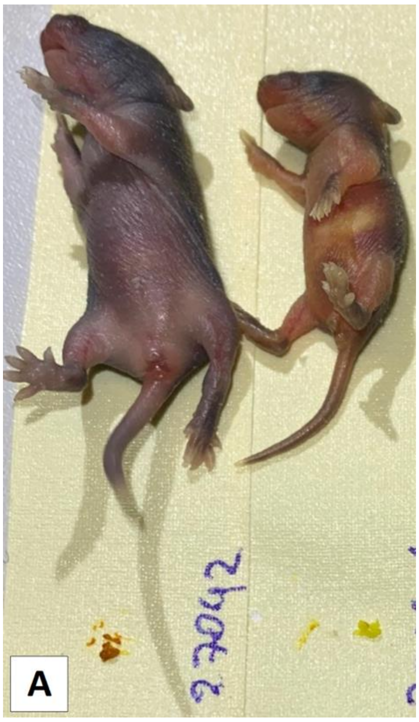

[A] Injection of Biliatresone induced clinical manifestations of BA in C57BL/6-neonates: jaundice, acholic stool and delayed weight gain (right) compared to control animal (left).

[B] Gross morphology of the EBDS Healthy. Control animal (left) compared to a Biliatresone treated neonate (right) showing twists and dilatation at the bile duct and enlarged gall bladder.

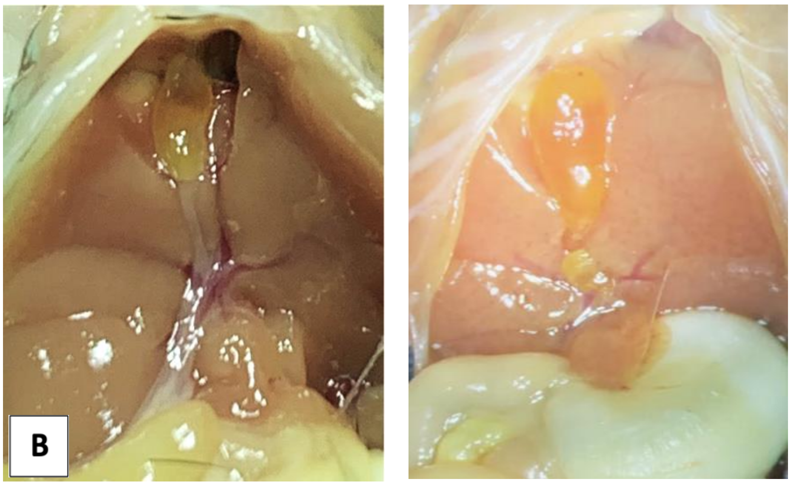

1. Sokol, R.J., et al., *Pathogenesis and outcome of biliary atresia: current concepts.* J Pediatr Gastroenterol Nutr, 2003. **37**(1): p. 4-21.
2. Ananth, R., *Neonatal Cholestasis: A Primer of Selected Etiologies.* Pediatr Ann, 2018. **47**(11): p. e433-e439.
3. Wehrman, A., O. Waisbourd-Zinman, and R.G. Wells, *Recent advances in understanding biliary atresia.* F1000Res, 2019. **8**.
4. Kassai, M., *Surgical treatment of biliary atresia.* Nihon Geka Gakkai Zasshi, 1983. **84**(9): p. 741-3.
5. Chardot, C., *Biliary atresia.* Orphanet J Rare Dis, 2006. **1**: p. 28.
6. Lakshminarayanan, B. and M. Davenport, *Biliary atresia: A comprehensive review.* J Autoimmun, 2016. **73**: p. 1-9.

7. Tiao, M.-M., et al., *Epidemiological features of biliary atresia in Taiwan, a national study 1996?2003.* Journal of Gastroenterology and Hepatology, 2007. **0**(0): p. 070827235605004.
8. Vic, P., et al., *[Biliary atresia in French Polynesia. Retrospective study of 10 years].* Arch Pediatr, 1994. **1**(7): p. 646-51.
9. Chiu, C.Y., et al., *Biliary atresia in preterm infants in Taiwan: a nationwide survey.* J Pediatr, 2013. **163**(1): p. 100-3.e1.
10. Chung, P.H.Y., S. Zheng, and P.K.H. Tam, *Biliary atresia: East versus west.* Semin Pediatr Surg, 2020. **29**(4): p. 150950.
11. Chardot, C., et al., *Improving outcomes of biliary atresia: French national series 1986-2009.* J Hepatol, 2013. **58**(6): p. 1209-17.
12. Livesey, E., et al., *Epidemiology of biliary atresia in England and Wales (1999-2006).* Arch Dis Child Fetal Neonatal Ed, 2009. **94**(6): p. F451-5.
13. Schreiber, R.A., et al., *Biliary atresia: the Canadian experience.* J Pediatr, 2007. **151**(6): p. 659-65, 665.e1.
14. Grizelj, R., et al., *Biliary atresia: the Croatian experience 1992–2006.* European Journal of Pediatrics, 2010. **169**(12): p. 1529-1534.
15. Wildhaber, B.E., et al., *Biliary atresia: Swiss national study, 1994-2004.* J Pediatr Gastroenterol Nutr, 2008. **46**(3): p. 299-307.
16. Muraji, T., D.L. Suskind, and N. Irie, *Biliary atresia: a new immunological insight into etiopathogenesis.* Expert Review of Gastroenterology & Hepatology, 2009. **3**(6): p. 599-606.
17. Mack, C.L. and R.J. Sokol, *Unraveling the Pathogenesis and Etiology of Biliary Atresia.* Pediatric Research, 2005. **57**(5): p. 87 - 94.
18. de Carvalho, E., C.A. Ivantes, and J.A. Bezerra, *Extrahepatic biliary atresia: current concepts and future directions.* J Pediatr (Rio J), 2007. **83**(2): p. 105-20.
19. Oetzmann von Sochaczewski, C., et al., *Experimentally Induced Biliary Atresia by Means of Rotavirus-Infection Is Directly Linked to Severe Damage of the Microvasculature in the Extrahepatic Bile Duct.* Anat Rec (Hoboken), 2019. **302**(5): p. 818-824.
20. Petersen, C., *Biliary atresia: the animal models.* Semin Pediatr Surg, 2012. **21**(3): p. 185-91.
21. Fried, S., et al., *Extrahepatic cholangiocyte obstruction is mediated by decreased glutathione, Wnt and Notch signaling pathways in a toxic model of biliary atresia.* Scientific Reports, 2020. **10**(1).
22. Koo, K.A., et al., *Reactivity of Biliatresone, a Natural Biliary Toxin, with Glutathione, Histamine, and Amino Acids.* Chem Res Toxicol, 2016. **29**(2): p. 142-9.
23. Yang, Y., et al., *The synthetic toxin biliatresone causes biliary atresia in mice.* Lab Invest, 2020. **100**(11): p. 1425-1435.
24. Schmidt, H.C., et al., *Extrahepatic Bile Duct and Gall Bladder Dissection in Nine-Day-Old Mouse Neonates.* J Vis Exp, 2022(186).

**Predictors for physical activity in schoolchildren after primary repair of esophageal atresia.**
ID: 216

**Kategorie: DGKCH - Kinderchirurgische Forschung**

Luisa Maria Frankenbach[1], Anne-Sophie Holler[2], Christina Oetzmann von Sochaczewski[3], Lucas Wessel[4], Anke Widenmann-Grolig[5], Tatjana Tamara König[1]

[1]*Universitätsmedizin Johannes Gutenberg-Universität, Klinik und Poliklinik für Kinderchirurgie, Mainz, Deutschland*, [2]*Klinikum der Universität München, LMU, Kinderchirurgische Klinik und Poliklinik im Dr. von Haunerschen Kinderspital, München, Deutschland*, [3]*Universitätsklinikum Bonn, Sektion Kinderchirurgie, Klinik und Poliklinik für Allgemein-, Viszeral-, Thorax- und Gefäßchirurgie, Bonn, Deutschland*, [4]*Medizinische Fakultät Mannheim der Universität Heidelberg, Klinik für Kinder- und Jugendchirurgie, Mannheim, Deutschland*, [5]*Selbsthilfeorganisation für Speiseröhrenerkrankungen KEKS e.V., Stuttgart, Deutschland*

**Background:**
Children born with esophageal atresia suffer from life-long gastrointestinal und pulmonary symptoms (1) and delayed weight gain and growth (2). There is evidence for impaired motor skills in early childhood (3) and lower muscle mass and exercise capacity in adolescence (4). The World Health Organization (WHO) recommends 60 minutes of daily physical activity for children and adolescents (5). The aim of this study was to identify factors that influence the amount of physical activity in children with uncomplicated esophaegal atresia.

**Materials and methods:**
In this multicentre study, EA patients were contacted by the participating centre or the patient organisation KEKS e.V. Data of 104 patients was reviewed. Only schoolchildren after primary esophageal repair were included in the analysis. The amount of physical activity in minutes was calculated according to the standardised “MoMo-Physical Activity Questionnaire”. The results were tested for normal distribution using the Kolmogorov-Smirnov-Test. Means with 95%-confidence intervals (95%-CI) were calculated, Spearman correlation, linear or multivariable linear regression were performed as appropriate.

**Results:**
Forty-six patients were included in the analysis (20 male, 26 female). The mean age was 10.7 years (standard deviation 3.3). All children participated in regular physical education in school, 43 had no restrictions for participation by their physician. The mean amount of physical activity was 227 minutes per week (95%-CI: 171-284), values were not normally distributed. In total, fifteen percent fulfilled the WHO recommendations for physical activity in children, 54 percent were currently member of a sports club. Several influencing factors on the amount of physical activity were analysed: weight-for-age and height-for-age at the age of four years (mean z-score weight -1.09, 95%-CI: -1.51; -0.68, mean z-score height -0.99, 95%-CI: -1.37; -0.61, $r^2$=0.007, p=0.32) or at the time of the survey (mean z-score weight -0,56, 95%-CI: -1.02; -0.11, mean z-score height -0.13, 95%-CI: -0.55; 0.30, $r^2$=0.04, p=0.16) had no influence. Furthermore, no correlation could be found for prematurity (n=24, r= 0.11, p=0.49), open (n=28) versus minimally invasive repair (n=13, r=0.35, p=0.83), congenital heart disease (total n=14, currently treated n=5, r=0.02, p=0.86), symptoms at rest (n= 15, r=-0.077, p=0.61) or symptoms during physical activity (n=12, r= -0.24, p=0.12).
The only factor significantly influencing the amount of physical activity in children was a father who was a member of a sports club (n=11, r=0.43, p=0,004). For other social factors, such as “father physically active” (n=26, r=0.12, p=0.46) “mother physically active” (n=23, r=-0.30, p=0.46), “mother member of a sports club” (n=14, r=0.27, p=0.065) there was no significant correlation.

**Conclusion:**
The amount of physical activity in schoolchildren with uncomplicated esophageal atresia depends on social rather than medical factors. Doctors should encourage children with esophageal atresia to be physically active on their own level independent of their medical history.

References
1. Dingemann C, Dietrich J, Zeidler J et al. Early complications after esophageal atresia repair: analysis of a German health insurance database covering a population of 8 million. Dis. Esophagus. 2016;29(7):780-6.
2. König TT, Stefanescu M-C, Wildermuth M et al. Perzentile für Körperlänge und -gewicht für Kinder mit Ösophagusatresie in Deutschland: eine Analyse von Registerdaten. Monatsschr Kinderheilkd. 2021(169):S 99.
3. König TT, Muensterer OJ. Physical Fitness and Locomotor Skills in Children With Esophageal Atresia-A Case Control Pilot Study. Front pediatr. 2018;6:337.
4. Arneitz C, Windhaber J, Castellani C, et al. Cardiorespiratory performance capacity and airway microbiome in patients following primary repair of esophageal atresia. Pediatr Res. 2021;90(1):66-73.
5. World Health Organization. Global Recommendations on Physical Activity for Health. WHO Press; 2010.

**Appendicitis manifests as two microbiome state types with oral pathogens influencing severity**
ID: 298

**Kategorie: DGKCH - Kinderchirurgische Forschung**

Georg Singer[1], Marcus Blohs[2], Mahnert Alexander[2], Kevin Brunnader[1], Christina Flucher[1], Christoph Castellani[1], Christine Moissl-Eichinger[2], Holger Till[1]

[1]*Department of Paediatric and Adolescent Surgery, Medical University of Graz, Graz, Österreich*, [2]*Diagnostic and Research Institute of Hygiene, Microbiology and Environmental Medicine, Medical University of Graz, Graz, Österreich*

**Background:**
Acute appendicitis (AA) is a spectrum ranging from uncomplicated appendicitis, which does not necessarily result in perforation to complicated appendicitis, which is associated with necrosis leading to perforation or periappendicular abscess. Even though AA is well-known to pediatric surgeons, little is known about the origins and etiopathogenesis of this disease, much less regarding the different disease types.

**Materials and methods:**
We investigated the microbiome (inter-domain amplicon and metagenome sequencing) of samples from the appendix, rectum and peritoneum of 60 children (median age 12, range 3 to 17 years) with AA to assess the composition and potential role of bacteria, archaea and fungi in local inflammation.

**Results:**
Histopathological investigation revealed subacute appendicitis in n=4, catarrhal appendicitis in n=14, phlegmonous in n=31, gangrenous/perforated in n=11 patients. The four patients without notable pathological findings (subacute samples) were excluded from further microbial analyses due to insufficient sample size. The analysis of the appendix microbial community revealed a shift depending on the severity of the AA. This shift was reflected by two major community state types that represented the complicated and uncomplicated cases. We could demonstrate that complicated, but not uncomplicated, appendicitis is associated with a significant local expansion of oral, bacterial pathogens in the appendix, most strongly influenced by necrotizing Fusobacterium spp., Porphyromonas and Parvimonas. Uncomplicated appendicitis, however, was characterised by gut-associated microbiomes. No significant associations were found between fungal taxa and archaeal and disease severity and any other clinical parameters.

**Conclusion:**

Our findings support the hypothesis that two disease types exist in AA, which cannot be distinguished using standard clinical characterization methods or by analysis of the patient’s rectal microbiome. An advanced microbiome diagnosis, however, could improve non-surgical treatment of uncomplicated AA.

**The immunomodulatory antimicrobial peptide LL-37 is upregulated in biliary atresia and contributes to hepatic damage and leucocyte recruitment**
ID: 427

**Kategorie: DGKCH - Kinderchirurgische Forschung**

Christoph Slavetinsky[1,2,3], Jule Basenach[2], Lena Schultheis[1], Silvio Nadalin[4], Stephan Singer[5], Andreas Peschel[3], Hans-Joachim Kirschner[1], Steven Warmann[1], Jörg Fuchs[1], Ekkehard Sturm[2]

[1]*Kinderchirurgie und -urologie , Universitätskinderklinik Tübingen, Tübingen, Deutschland*, [2]*Kindergastroenterologie und -hepatologie, Universitätskinderklinik Tübingen, Tübingen, Deutschland*, [3]*Infektionsbiologie , Interfakultäres Institut für Mikrobiologie und Infektionsmedizin Tübingen, Tübingen, Deutschland*, [4]*Universitätsklinik für Allgemein-, Viszeral- und Transplantationschirurgie, Tübingen, Deutschland*, [5]*Allgemeine und Molekulare Pathologie und Pathologische Anatomie Tübingen, Tübingen, Deutschland*

**Background:**
Biliary atresia (BA) is life-threatening liver disease and characterized by progressive liver inflammation, fibro-obliterative obstruction of bile ducts and subsequent liver fibrosis/cirrhosis. Despite an unclear pathogenesis, recent studies favor an etiology through microbe- or toxin-triggered (auto-)immune dysregulation in liver and bile ducts. Liver fibrosis of BA is characterized by a massive leucocyte infiltration and dysregulation but also a damage microcirculation. Antimicrobial peptides (AMPs) belong to the first line of innate immunity against invading microbes and possess regulatory functions to control inflammation and immune response (e.g., by chemoattraction). The role of AMPs in hepatic physiology and pathophysiology in children and their impact on liver inflammation is unknown.
We aimed to characterize expression of antimicrobial peptides and proteins in BA compared to normal and disease control liver tissue, to associate expression patterns with clinical course and outcome, and to assess their impact on liver inflammation and leucocyte trafficking.

**Materials and methods:**
Gene expression of the antimicrobial peptides and proteins LL-37, LEAP-1, Lysozyme, and MBL were analyzed by qPCR from liver tissue of patients with BA at early (eBA; biopsy at diagnosis; n = 19) and late (lBA; biopsy at liver transplantation; n = 30) liver disease stages, both compared to age-matched normal liver tissue derived from cancer patients (n = 10), and tissue from familial cholestatic disorders (Progressive familial intrahepatic cholestasis (PFIC) and Alagille syndrome (ALGS) at early (eFCD; n = 11) and late (lFCD; n = 6) liver disease stages. Expressions were analyzed for native liver survival by Kaplan–Meier estimator, clearance of jaundice after Kasai portoenterostomy (KPE), matched with age at biopsy/disease state, and correlated with pro-fibrotic activity (TGF-ß expression) and cholestasis (serum bilirubin) by regression analysis. LL-37 expression in BA liver sections was localized by immunohistochemistry and co-localized with ICAM-I and VCAM-I by immunofluorescence.

Hepatocytes (HEPG2), primary endothelial cells (human umbilical vein endothelial cells, HUVECs) and smooth muscle cells (human umbilical artery smooth muscle cells, HUASMCs) treated with LL-37 were assessed for cell viability by MTT assay and for apoptosis by FACS-based *Annexin V* binding *assay. Upregulation of ICAM-1 and VCAM-I expression upon LL-37 treatment of endothelial cells was measured by qPCR and immunofluorescence.* Group comparison was made by one-way ANOVA (*, $P < 0.05$; **, $P < 0.01$; ***, $P < 0.001$; ns, not significant).

**Results:**

Liver gene expression of LL-37 but not of LEAP-1, Lysozyme and MBL is significantly upregulated in early BA compared to normal and disease controls. Age (in days) at biopsy does not affect LL-37 expression in early BA. Both LL-37 and LEAP-1 expression are significantly downregulated during course of disease (late BA). LL-37 expression in early BA does correlate with TGF-ß expression (profibrotic activity marker) ($R^2 = 0.44$, $P = 0.02$) but not with bilirubin serum levels (cholestasis marker) ($R^2 = 0.02$, $P = 0.59$). An increased LL-37 expression in early BA does not significantly affect native liver survival or clearance of jaundice after KPE. Immunohistochemistry of BA liver sections localizes LL-37 expression in immune cells, artery smooth muscle cells and venous endothelial cells. LL-37 has an *in vitro* dose-dependent cytotoxic effect on both primary endothelial and smooth muscle cells and *induces apoptosis in endothelial cells. Further, the endothelial leucocyte adhesion molecules ICAM-1 and VCAM-1, known to be upregulated by endothelia upon inflammatory stress, showed to be dose-dependently upregulated by LL-37 in endothelial cells and co-localized* with LL-37 in immunofluorescence from liver sections of BA.

**Conclusion:**

Antimicrobial peptides and proteins are expressed in normal and diseased pediatric liver tissue. Expression of LL-37 is upregulated in early BA and downregulated in late BA, which may allow discrimination between disease states. Increased early expression of LL-37 is associated with increased expression of the profibrotic activity marker TGF-ß, but does not predict native liver survival. LL-37 expression in BA was localized in immune cells and hepatic vasculature. *In vitro* treatment with LL-37 was cytotoxic for endothelial cells and upregulated the adhesion molecules ICAM-1 and VCAM-1, which co-localized *in vivo* with LL-37 in BA liver sections. These proapoptotic effects may contribute to microcirculation defects in BA, while upregulation of adhesion molecules could promote hepatic leucocyte infiltration. LL-37 may act as modulator of cell damage, inflammation and immune activation in BA resulting in early progression of disease.

**Invasive Staphylococcus capitis isolates from necrotizing enterocolitis and neonatal sepsis specified to resist human intestinal gut immunity and opposing colonizers**
ID: 435

**Kategorie: DGKCH - Kinderchirurgische Forschung**

Christoph Slavetinsky[1,2], Lara Kränkel[2], Janna Hauser[2], Jeffrey Power[2], Annika Schmidt[3], Steven Warmann[1], Christian Gille[4], Andreas Peschel[2], Jörg Fuchs[1]

[1]*Kinderchirurgie und -urologie, Universitätskinderklinik Tübingen, Tübingen, Deutschland*, [2]*Infektionsbiologie, Interfakultäres Institut für Mikrobiologie und Infektionsmedizin Tübingen, Tübingen, Deutschland*, [3]*Medizinische Mikrobiologie und Hygiene Tübingen, Tübingen, Deutschland*, [4]*Neonatologie, Universitätskinderklinik Heidelberg, Heidelberg, Deutschland*

**Background:**
Necrotizing enterocolitis (NEC) and neonatal Late-Onset-Sepsis (LOS) are major causes of neonatal morbidity and mortality, especially in preterm infants with very low birth weight (VLBW). NEC´s pathophysiology remains unclear, but is characterized by a leaky epithelium often combined with bacterial invasion and subsequent gut inflammation and necrosis. Preterm infants possess a highly vulnerable gut immune barrier subjected to a premature microbial colonization. Intestinal dysbiosis is both a risk factor for NEC and one major route of infection in LOS. In contrast to adult sepsis, LOS is most commonly (>50%) caused by coagulase-negative staphylococci (CoNS) with high rates of *Staphylococcus capitis* isolates. CoNS are the first and most prevalent colonizers of the newborn's skin and mucosa (e.g., intestinal tract). A multi-resistant *S. capitis* clone called NRCS-A has recently caused endemic outbreaks of bloodstream infections in neonatal intensive care units (NICU) in various countries.
We established a collection of CoNS *(S. capitis*, *S. epidermidis* and *S. haemolyticus)* isolated from NEC (intraoperative swab & blood culture) and LOS (blood culture) in our NICU to assess genetical and phenotypical characteristics of such strains allowing to specify for the neonatal (intestinal) niche.

**Materials and methods:**
CoNS isolates (*S. capitis*, n = 14; *S. epidermidis*, n = 9; *S. haemolyticus*, n = 4) were sequenced (whole genome sequencing (WGS)) by next-generation sequencing (NGS) technology using libraries for Illumina short-read sequencing prepared with the Illumina Nextera™ DNA Flex Library Preparation Kit.
Minimal inhibitory concentrations (MIC) of antimicrobials were analyzed by MIC test strips (Liofilchem) or by broth microdilution. Bacterial cell wall thickness was determined by transmission electron microscopy (TEM) of bacterial cells with six thickness measurements per cell. Binding to immunoglobulins / MBL was determined by incubation of respective bacterial strains in increasing concentrations IgG, IgA or MBL and subsequent quantification by means of secondary antibodies in flow cytometry. Group comparison was made by one-way ANOVA or student´s t test (*, $P < 0.05$; **, $P < 0.01$; ***, $P < 0.001$; ****, $P < 0.0001$; ns, not significant).

**Results:**
Whole genome sequencing assigns our *S. capitis* NEC and LOS isolates to the NRCS-A clonage and reveals the presence of exclusive genes only present in the invasive CoNS isolates compared to non-invasive strains. All invasive isolates exclusively express the nisin resistance gene (*nsr*) and a *tarIJL* gene cluster crucial for synthesis of *S. aureus*-specific non-proteinaceous adhesion factors Wall Teichoic Acids (WTA). Of note, most isolates possess a premature stop codon in *tarJ* arguing against a crucial function of this operon. All isolates express a SCC*mec* element mediating methicillin resistance in all these multi-resistant isolates. The *S. capitis* isolates revealed an increased cell wall thickness compared to non-invasive strains ($P < 0.0001$), a major phenotypic characteristic of highly antibiotic resistant gram-positive bacteria and sign for an altered cell wall. Consistent with the expression of *nsr*, our isolates revealed an increased minimal inhibitory concentration (MIC) for the bacteriocin nisin ($P < 0.0001$), likely to support overgrowth in the establishing gut flora against nisin-producing intestinal colonizers such as lactobacilli. To assess whether the invasive CoNS isolates are also resistant to factors of the human (intestinal) immune system, we analyzed resistance to antimicrobial peptides and bacterial binding to immunoglobulins. Our isolates show a decreased susceptibility to human alpha-defensin 5 (HD5) ($P < 0.0001$), the major antimicrobial peptide controlling microbial homeostasis in the human gut, but not to HD6 and LL-37. FACS binding analysis to IgA, IgG and MBL revealed an altered binding of *S. capitis* and *S. haemolyticus* bloodstream isolates compared to non-invasive strains. While both serum and colostrum IgA deposition on those strains was decreased, MBL and IgG deposition was increased in bloodstream isolates. IgA is actively secreted into intestinal fluid, while MBL and IgG rather serve for (epithelial) cell protection. Evasion from IgA binding could serve to support intestinal overgrowth of such strains to subsequently invade through a leaky gut barrier.

**Conclusion:**
CoNS isolates from NEC and LOS possess specific genetic and phenotypic changes to adapt for successful intrusion into the neonatal gut microbiome and subsequent host invasion. Increased resistance of these invasive isolates enables to withstand opposing colonizers, human intestinal immune factors and antibiotics. A specific immunoglobulin binding pattern of our isolates could allow increased intestinal growth but is more likely to be recognized and bound in serum, a typical characteristic of more invasive bacteria (such as *S. aureus*). Further experiments are required to uncover the underlying mechanisms leading to increased virulence for intestinal wall and bloodstream invasion. Specific detection and treatment of high-risk bacteria for neonates, which are often overlooked due to a limited relevance for adult health, is required to improve health care for preterm and newborn infants.

**Isolation of human myenteric plexus - a different approach to enteric neurospheres**

ID: 469

**Kategorie: DGKCH - Kinderchirurgische Forschung**

Pauline Schuppert[1], Johanna Hagens[1], Hans Christian Schmidt[1], Laia Pagerols Raluy[1], Magdalena Trochimiuk[1], Birgit Appl[1], Konrad Reinshagen[1], Christian Tomuschat[1]

[1]*University Medical Center Hamburg-Eppendorf, Department of Pediatric Surgery, Hamburg, Deutschland*

**Background:**

Neurospheres are free-floating 3D-cell clusters of neural stem and progenitor cells. They were originally discovered following the isolation and culture of neural cells from murine brain tissue and have since then been grown from various other tissues, including the gut. [1,2] Enteric neurospheres have mainly been studied in mouse models – particularly focusing on their therapeutic potential in animals expressing enteric nervous system (ENS) pathologies. [3]

The traditional method for the generation of human enteric neurospheres requires the use of collagenase, dispase and repetitive trituration for tissue dissociation, followed by filtration using cell strainers. [4]

It has been criticized that neurospheres acquired by this method should rather be termed enterospheres, as they contain significant amounts of non-neural cells. In order to acquire neurospheres selectively composed of neural cells, FACS is feasible but does require time and a FACS-trained researcher. [4]

Thus, the authors propose an alternative approach for the generation of human enteric neurospheres.

**Materials and methods:**

The authors modified a protocol by Grundmann et al. originally developed and used for the generation of neurospheres from adult mice, which focuses on the isolation of myenteric plexus (MP) networks utilizing a highly purified collagenase. [5]

Following ethical approval, the tunica muscularis was isolated from human gut samples via microdissection, cut into smaller pieces and incubated overnight with a purified collagenase. As the ENS does not contain collagen, overnight digestion and two additional mechanical disruption steps led to the isolation of numerous MP networks (Fig. 1), which were cultured in proliferation media supplemented with growth factors. The media was changed every three days whereas growth factors were supplemented daily.

**Results:**

Isolated myenteric plexus started forming cell clusters after 24 hours. Membranes were observed during the first two days in culture and fully developed neurospheres were ready for harvesting after 72 hours. (Fig. 2)

**Conclusion:**

The authors were able to modify a protocol by Grundmann et al. for the isolation of myenteric plexus from human gut tissue with high consistency between samples. Neurospheres and their supernatant were processed for further experiments including ELISA and qPCR.

The proposed method is straightforward and therefore especially beneficial for researchers unskilled in primary tissue handling. Moreover, it has been proven to offer a significantly higher yield of neural cells compared to the traditional technique in a mouse model, as well as providing viable MP networks without contamination by surrounding cells. [5] Considering the limited availability of human gut tissue samples in comparison to animal-derived samples, this technique might prove advantageous to researchers conducting experiments where a high yield of tissue is required.

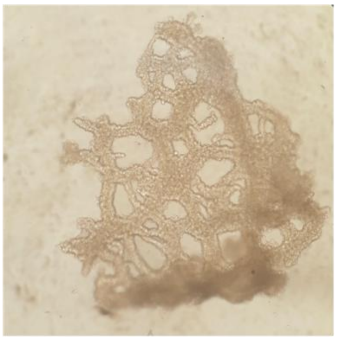

Fig. 1 – Myenteric plexus

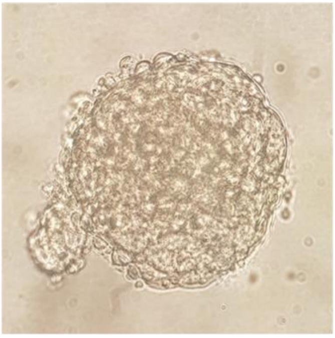

Fig. 2 – Neurosphere after 72h in culture

**References:**

1. Reynolds BA, Weiss S. Generation of neurons and astrocytes from isolated cells of the adult mammalian central nervous system. Science. 1992 Mar 27;255(5052):1707-10. doi: 10.1126/science.1553558. PMID: 1553558.
2. Rauch U, Hänsgen A, Hagl C, Holland-Cunz S, Schäfer KH. Isolation and cultivation of neuronal precursor cells from the developing human enteric nervous system as a tool for cell therapy in dysganglionosis. Int J Colorectal Dis. 2006 Sep;21(6):554-9. doi: 10.1007/s00384-005-0051-z. Epub 2005 Nov 3. PMID: 16267668.
3. McCann CJ, Cooper JE, Natarajan D, Jevans B, Burnett LE, Burns AJ, Thapar N. Transplantation of enteric nervous system stem cells rescues nitric oxide synthase deficient mouse colon. Nat Commun. 2017 Jul 3;8:15937. doi: 10.1038/ncomms15937. PMID: 28671186; PMCID: PMC5500880.

4. Neckel PH, Scharr M, Seid K, Nothelfer K, Fuchs J, Obermayr F, Hirt B, Huber SM, Just L. Wnt Receptor Frizzled-4 as a Marker for Isolation of Enteric Neural Progenitors in Human Children. Cells. 2019 Jul 30;8(8):792. doi: 10.3390/cells8080792. PMID: 31366044; PMCID: PMC6721585.
5. Grundmann D, Klotz M, Rabe H, Glanemann M, Schäfer KH. Isolation of high-purity myenteric plexus from adult human and mouse gastrointestinal tract. Sci Rep. 2015 Mar 20;5:9226. doi: 10.1038/srep09226. PMID: 25791532; PMCID: PMC4366762.

### Appendicitis in children with a ventriculo-peritoneal shunt

ID: 503

**Kategorie: DGKCH - Kinderchirurgische Forschung**

Glenn Malin Fröschle[1], Johanna Hagens[1], Konrad Reinshagen[1], Christian Tomuschat[1]

[1]*Universitätsklinikum Hamburg-Eppendorf, Klinik für Kinderchirurgie , Hamburg, Deutschland*

**Background:**

Abdominal shunt related problems are common in patients with ventriculo-peritoneal shunt (VPS). These patients can be difficult to diagnose if they present with fever, vomiting and abdominal discomfort, particularly in the lower quadrant in addition to increased signs of inflammation. When a child with a VPS presents with clinical symptoms of an "acute abdomen," the doctor is sometimes faced with a clinical challenge, as a primary intra-abdominal shunt-independent disease must be distinguished from shunt-related infection [1]. Appendicitis, primary peritonitis, adhesive small-bowel obstruction, or an infected abdominal pseudocyst are usually included in the differential diagnosis [2]. There are only a limited number of cases reported in the literature of children and adults with VPS being managed for appendicitis. The purpose of the current study was to identify potential risk factors and to outline our management of patients with appendicitis and ventriculoperitoneal shunts (VPS).

**Materials and methods:**

In our retrospective study we analysed all paediatric patients with VPS who presented with an acute abdomen between 2012 and 2022 at a tertiary-care paediatric facility. Patients were divided into two groups based on their diagnoses: group A (appendicitis) and group B (primary peritonitis). Medical records were examined to further look at the diagnostic interventions and results, operative approach, complications, shunt exteriorization, peritoneal culture, liquor culture and length of the antibiotic treatment. In addition, information on length of stay (LOS) and long-term outcome was acquired.

**Results:**

We could include a total of 17 patients between June 2012 and May 2022 who had clinical signs of acute abdominal discomfort or acute abdomen. We could identify 7 patients with a histopathological appendicitis and 10 patients with primary peritonitis.

The median age at initial shunt placement was 7.5 months in the appendicitis group and 4.5 months in the peritonitis group. Prior to the beginning of abdominal pain, the median shunt duration was 4 months in the appendicitis group and was 5 months in the peritonitis group.

We were able to show that the median age at surgery in the appendicitis group was 34 months compared to a median age at surgery of 71 months in the peritonitis group, showing that the children in the appendicitis group were younger. The children in the appendicitis group presented clinically sicker and had more often neurological symptoms.

In the appendicitis group the preoperative CRP (mg/dl) was 152 (range, 25 mg/dl – 363 mg/dl) and the median temperature at presentation was 39°C (range, 37.7°C - 40.7°C) compared to a preoperative CRP (mg/dl) of 104 (range, 0 mg/dl – 305 mg/dl) and a median temperature at presentation of 38.4°C (range, 36.7°C - 39°C) in the peritonitis group (no statistical significance). In 85% of patients in the appendicitis group the abdominal cultures were positive. We could only find an abdominal positive culture in 40% in the peritonitis group. All patients with appendicitis had their VPS exteriorized.

**Conclusion:**

Appendicitis in patients is a rare disease with just few literatures describing the optimal diagnostic tools and treatment in patients with VPS presenting with abdominal pain and signs of infection. It still is not easy to diagnose appendicitis in patients with VPS and to distinguish between those pediatric patients with VPS who should be operated and those who should be treated conservatively.

We could find out that the median age of the patients with VPS and appendicitis on diagnosis (group A) was 34 months (2.8 years) compared to 71 months (5.9 years) in group B. This shows an important difference in age between the two groups. The paediatric surgery community is aware that toddlers and very young children are frequently neglected when it comes to appendicitis; as a result, the perforation rate is higher and the clinical course more severe in these patients [3]. Typically, the classic abdominal symptoms in this age group are vomiting, pain, fever and diarrhoea and have been all present in both groups [3]. Additionally, to the abdominal symptoms, sometimes patients with VPS display neurological symptoms. The onset of new neurological symptoms in a child with VPS is usually indicative for a shunt dysfunction [4]. In addition to ruling out a proximal dysfunction, which is by far the most common reason for shunt failure, a distal malfunction caused by obstruction of the catheter's distal tip should be ruled out. Despite the full array of diagnostic tools, including high-resolution ultrasonography (US), the diagnosis is still difficult, which could postpone a crucial operation and increase the risk of an appendix rupture, as demonstrated in the current cohort [2,5].The median time from the onset of symptoms to operation or final diagnosis in the present cohort was three days for group A and 4.5 days for group B, making it simple to understand, that often a “diagnostic loop” may delay the proper diagnosis.

The median interval in group A for shunt reinsertion was 20 days and group B 14 days. However, it is controversial whether the catheter's distal end should be reinserted in the peritoneum or implanted, preferably into the atrium of the heart to prevent a second abdominal approach. In our case series, each catheter was reinserted into the peritoneal cavity. We had a moderate revision rate within one year (group A 28.6% vs. group B 40%) in both groups, although it is questionable and impossible to prove in the current study that reinsertion into the abdominal cavity is related to consecutive revision surgery.

We conclude that surgery should be aggressively administered to patients who have both appendicitis and a VPS. In those with evident perforation, shunt exteriorization is suggested. Shunt infection and dysfunction should be ruled out in patients who only have peritonitis, and surgical care should be initiated depending on the severity of the symptoms.

### References

[1] Häussler B, Menardi G, Hausberger K, Hager J. Ventriculoperitoneal shunt infection and appendicitis in children. European Journal of Paediatric Surgery 2001.

[2] Ein SH, Miller S, Rutka JT. Appendicitis in the child with a ventriculo-peritoneal shunt: a 30-year review. J Pediatr Surg 2006;41:1255–8. https://doi.org/10.1016/j.jpedsurg.2006.03.004.

[3] Almaramhy HH. Acute appendicitis in young children less than 5 years: review article. Ital J Pediatr 2017;43:15. https://doi.org/10.1186/s13052-017-0335-2.

[4] Ein SH, Miller S, Rutka JT. Appendicitis in the child with a ventriculo-peritoneal shunt: a 30-year review. J Pediatr Surg 2006;41. https://doi.org/10.1016/j.jpedsurg.2006.03.004.

[5] Dalfino JC, Adamo MA, Gandhi RH, Boulos AS, Waldm An JB. Conservative management of ventriculoperitoneal shunts in the setting of abdominal and pelvic infections: Clinical article. J Neurosurg Pediatr 2012;9:69–72. https://doi.org/10.3171/2011.10.PEDS1189.

**NEC in a Dish – Establishment of an Organoid Co-Culture with Neutrophils and their possible role in NEC pathophysiology**

ID: 569

**Kategorie: DGKCH - Kinderchirurgische Forschung**

Deirdre Vincent[1], Kim Heuer[1], Laia Pagerols Raluy[1], Johanna Hagens[1], Jan Kolman[1], Jasmin Wesche[2], Konrad Reinshagen[1], Michael Boettcher[3]

[1]*Universitätsklinikum Hamburg-Eppendorf, Kinderchirurgie, Hamburg, Deutschland*, [2]*Leibniz-Institut für Virologie, Virus Immunologie, Hamburg, Deutschland*, [3]*Universitätsmedizin Mannheim, Universität Heidelberg, Kinderchirurgie, Mannheim, Deutschland*

**Background:**

Despite being one of the leading causes of neonatal death and long-term morbidity, necrotizing enterocolitis (NEC) pathophysiology remains inconclusive. While it has been shown that NEC development involves a combination of bowel and immune system prematurity, abnormal bacterial colonization, and ischemia-reperfusion injury, which is partially mediated by neutrophils, development of targeted NEC treatment options is lagging, mainly due to limitations of current NEC animal testing models. Therefore, this study aimed to develop an organoid NEC model to understand NEC pathophysiology, validate neutrophils' role in NEC, and bridge the gap between animal and human NEC research.

**Materials and methods:**

Organoid cultures were established from human neonatal intestinal samples with (1) NEC or (2) without gut inflammation (controls – CO). Culture subsets were treated with lipopolysaccharide for 48 hours and/or co-cultured with neutrophils for 24 hours. Neutrophil survival (PI / Annexin) and activation (CD11b / CD66b), as well as co-cultures' (neutrophils and organoids) TLR-4 expression and cell death marker (zombies) were analyzed using flow cytometry. (1) Organoids' NEC status, measured using a NEC score, and (2) neutrophil recruitment, were analyzed using immunofluorescence.

**Results:**

16 patients (NEC 8; CO 8) were included, with NEC neonates being significantly younger than COs ($p$ = 0.005). While clinical and demographic parameters did not differ significantly between groups, TLR-4 expression after LPS administration was significantly higher in NEC organoids. Also, markers of intestinal apoptosis and NEC scores were significantly increased in NEC versus CO organoids. Differences were more pronounced with the addition of neutrophils. With respect to neutrophil activation, markers were increased when cultured with organoids, while their TLR-4 expression was higher when cultured with NEC organoids specifically.

**Conclusion:**

Our study suggests that an increased innate TLR-4 expression might cause a genetic susceptibility for NEC development. Moreover, stimulation of the TLR-4 receptor using LPS results in an exacerbated response and more NEC-like lesions in NEC organoids, which worsens when neutrophils are added. Thus, in line with previous animal NEC study results, organoid co-cultures demonstrated that neutrophils play an essential role in NEC manifestation and maintenance. Furthermore, it can be concluded that NEC pathomechanisms can also be tested using the NEC in a dish model.

**Mechanical compression in experimental congenital diaphragmatic hernia is associated with an increase of macrophage density**
ID: 641

**Kategorie: DGKCH - Kinderchirurgische Forschung**

Fabian Doktor[1], Rebeca Lopes Figueira[1], Kasra Khalaj[1], Matisse Blundell[1], Mikal Obed[1], Martin Lacher[2], Augusto Zani[1]

[1]*The Hospital for Sick Children, Division of General and Thoracic Surgery, Toronto, Canada*,
[2]*University of Leipzig, Department of Pediatric Surgery, Leipzig, Deutschland*

**Background:**
Congenital diaphragmatic hernia (CDH) is a birth defect characterized by fetal pulmonary hypoplasia, a condition that remains one of the main determinants of the high morbidity and mortality rates in this patient population. We have recently shown that CDH lungs have an inflammatory signature with a high macrophage density. However, the cause for macrophage recruitment to the fetal lung is unknown. Herein, we investigated whether mechanical compression contributes to the increase in the macrophage enrichment that is found in CDH lungs.

**Materials and methods:**
To explore the role of mechanical compression, we compared two models of pulmonary hypoplasia in fetal rats: CDH (compression) vs. oligohydramnios (no compression). *CDH model*: pulmonary hypoplasia was induced by administration of nitrofen to dams on embryonic day (E) 9.5, which caused CDH in ~50% of pups. In this study, only pups with confirmed CDH were enrolled. Controls were from dams that received olive oil on E9.5.
*Oligohydramnios model*: pulmonary hypoplasia was induced by aspiration of amniotic fluid (150uL) with a 27-gauge needle syringe on E16.5. Pups that were not subjected to amniotic fluid aspiration served as controls.
*Outcome measures*: Fetal lungs were harvested and compared within their groups for:
1) severity of hypoplasia measured as airspace density (hematoxylin & eosin) by mean linear intercept (MLI) and radial alveolar count (RAC);
2) macrophage density by CD68 immunofluorescence staining.
*Statistics*: Mann-Whitney and unpaired t-test were used as appropriate. Data are presented as mean ± SD.

**Results:**
1) Pulmonary hypoplasia was confirmed in both models. Both CDH and oligohydramnios lungs had an increased MLI (CDH: 69.5 ± 16.5 vs. control 21.9 ± 4.8, p<0.0001; oligohydramnios: 84.2 ± 15.8 vs.

control 56.3 ± 15.8, p=0.003) and a decreased RAC (CDH: 2.4 ± 0.5 vs. control 4 ± 1.1, p<0.0001; oligohydramnios: 0.78 ± 0.5 vs. control 1.27 ± 0.5, p=0.019).
2) Macrophage density: only CDH lungs were enriched with macrophages. Compared to controls, CD68 expression was increased in CDH lungs (CDH: 9.7 ± 4.9 vs. control 1.2 ± 1.5, p<0.0001), but not in oligohydramnios lungs (oligohydramnios: 6.2 ± 2.65 vs. control 6.4 ± 3.3, p=0.76).

**Conclusion:**
This study confirms that fetal hypoplastic lungs that are subjected to external mechanical compression have high macrophage enrichment. This observation is in line with studies in other organs that have reported inflammation with macrophage involvement following changes in tissue-specific mechanical properties. Further studies are needed to unravel the mechanism that modulates macrophage recruitment to the fetal lung.

**Localization of AQP1 expression in Hirschsprung`s Disease and its possible implications**
ID: 835

**Kategorie: DGKCH - Kinderchirurgische Forschung**

Rebecca Angresius[1], Zihe Hou[1], Natasha Whitehead[1], Stefan Holland-Cunz[1], Stefanie Gros[1]

[1]*Universitäts-Kinderspital beider Basel (UKBB), Basel, Schweiz*

**Background:**
Hirschsprung`s Disease (HD) is a congenital intestinal disorder characterized by a variable length of distal colonic aganglionosis. It is caused by aberrant processes of the enteric neural crest-derived cells that could include proliferation, migration, differentiation, and survival. Enterocolitis is a potentially life-threatening complication. Aquaporin 1 (AQP1) is physiologically expressed in several parts of the intestine including mucosa, submucosal lamina, the capillary endothelial cells and in the enteric nervous system. Its function in each anatomical structure remains not clearly defined. The aim of the study was to characterize the AQP1 expression in Hirschsprung`s Disease and correlate its expression with severity of disease including the risk for enterocolitis.

**Materials and methods:**
Tissue samples from 20 patients with Hirschsprung`s disease were examined by immunohistochemical and immunofluorescence staining and compared with control tissue. Co-staining against AQP1, β3 tubulin and AChE was carried out. Whole slide scanning was performed and AQP1, β3 tubulin and AChE expression was analysed visually regarding its anatomical expression pattern and by threshold image analysis.

**Results:**
An increase of AQP1 expression was recognized in the distal part of the affected colon tissue in Hirschsprung patients compared with control tissue in correlation with β3 tubulin and AChE expression. While AQP1 is known to be physiologically expressed in vascular endothelial cells and in ganglia of the enteric nervous system, in these patients this particular localization is missing, however we found an increased expression in the mucosa.

**Conclusion:**
A distinct pattern of AQP1 expression could be observed in the colon of patients with Hirschsprung`s Disease, suggesting an imbalance in membrane water permeability, which might directly impact on development of enterocolitis, as well as hinting towards a regulatory role of AQP1 in the enteric nervous system.

**Hitting different – Differentiation of intestinal colonic organoids influences the intestinal barrier function under proinflammatory conditions.**

ID: 879

**Kategorie: DGKCH - Kinderchirurgische Forschung**

Johanna Hagens[1], Pauline Schuppert[1], Kim Marili Heuer[1], Clara Isabell Philippi[1], Hans Christian Schmidt[1], Laia Pagerols Raluy[1], Konrad Reinshagen[1], Christian Tomuschat[1]

[1]*University Medical Center Hamburg-Eppendorf, Department of Pediatric Surgery, Hamburg, Deutschland*

**Background:**

The human intestinal mucosa comprises a bright variety of highly specialized cell types [1]. Enteroids, which may be produced from either pluripotent (PSC) or adult stem cells (ASC), constitute a new benchmark model for the study of intestinal diseases. While PSCs retain fetal-like, immature features, ASCs cultivated from intestinal crypt tissue can be expanded in their stem cell-like condition or differentiated into a fully functional mucosal layer while preserving their donor-specific epigenetic signature [2]. As they migrate to the luminal surface after development from stem cells at the bottom of the mucosal crypts, intestinal cells balance physiological apoptosis with epithelial renewal *in vivo* [3, 4]. Tight and adhesive junctions that bridge the intercellular gaps are primarily responsible for maintaining the intestinal barrier homeostasis in this process [5]. Interestingly, many publications don’t specify whether or not their ASC enteroids have undergone a differentiation process, challenging translation of the results into clinical context. Therefore, the purpose of this work was to assess possible differences between differentiated and undifferentiated cultures in the response of ASC intestinal colonic organoids to proinflammatory stimuli indicated by intestinal barrier integrity.

**Materials and methods:**

Intestinal colonic organoids derived from human ganglionic Hirschsprung (gHIO, n = 9) and healthy control samples (CIO, n = 9) were either cultured in standard growth medium for stem cell preservation or differentiated using an intestinal-specific differentiation medium. Enteroids were stimulated with a proinflammatory cytokine cocktail containing human TNFα, IL-1β, and IL-6 to simulate an intestinal inflammation. Acute stimulation was defined as a 72-hours treatment with daily medium exchange. Chronic proinflammatory conditions were simulated with a 96-hour treatment and medium changes every two days. Intestinal barrier marker expressions were quantified in Western Blot (WB). Confocal immunofluorescence microscopy (cIF) and a FITC-dextran permeability assay were used to assess intestinal barrier function. Results were analysed using ImageJ and GraphPad Prism.

**Results:**

All cultures showed an increase in E-Cadherin, Occludin and Claudin-3 following differentiation without additional stimulation. In stem cell-like cultures, acute proinflammatory stimulation led to an increase in E-Cadherin, Occludin and Claudin-3, whereas differentiated enteroids revealed a controversial decrease in Occludin and Claudin-3 for gHIOs and Claudin-3 for CIOs. Undifferentiated enteroids exhibited only slight variations of barrier marker expression under chronic proinflammatory circumstances, but differentiated cultures responded with significant alterations in barrier protein expression, particularly in CIOs. All studied barrier proteins in CIOs decreased after chronic proinflammatory stimulation, whereas protein levels in gHIOs were almost steady. In comparison to CIOs, gHIOs generally expressed lower amounts of E-Cadherin, Occludin and Claudin-3. cIF and FITC-dextran-assay detected corresponding results with barrier impairment in differentiated cultures after acute proinflammatory stimulation.

**Conclusion:**

Differentiation of ASC intestinal colonic organoids reveals a relevant impact on the intestinal barrier homeostasis under proinflammatory conditions. While acute proinflammatory stimulation causes an upregulation of barrier proteins in undifferentiated cultures, specialized cells preferably react with barrier disruption. Changes through chronic stimulation are more pronounced in differentiated enteroids compared to the stem cell cultures. This emphasizes the need of differentiation in ASC cultures to accurately represent the *in vivo* scenario, since healthy controls appear to be particularly prone to proinflammatory stimulation after differentiation but not before.

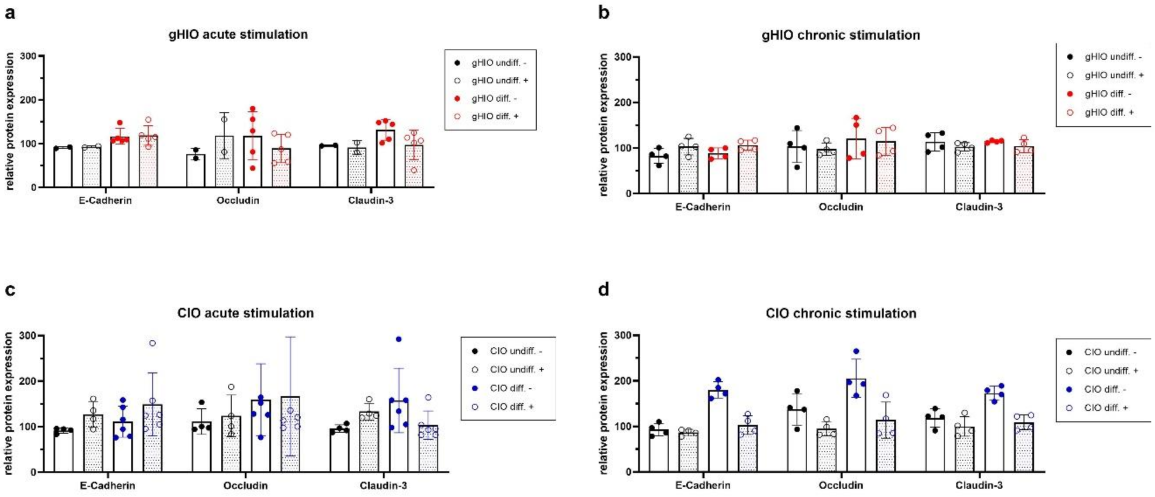

**Figure 1** Relative protein expression of the intestinal barrier markers E-Cadherin, Occludin and Claudin-3 in Western Blot analysis in different culture origins and treatment conditions. Undifferentiated cultures presented in black. Differentiated cultures highlighted in red (gHIO) and blue (CIO). (**a**) Acute proinflammatory stimulation in gHIOs. (**b**) Chronic proinflammatory stimulation in gHIOs. (**c**) Acute proinflammatory stimulation in CIOs. (**d**) Chronic proinflammatory stimulation in CIOs.

**References:**

1. Kong S, Zhang YH, Zhang W. *Regulation of Intestinal Epithelial Cells Properties and Functions by Amino Acids*. Biomed Res Int. 2018 May 9;**2018**:2819154. doi: 10.1155/2018/2819154.
2. Funata M, Nio Y, Erion DM, Thompson WL, Takebe T. *The promise of human organoids in the digestive system*. Cell Death Differ. 2021 Jan;**28**(1):84-94. doi: 10.1038/s41418-020-00661-3. Epub 2020 Nov 17.
3. Chelakkot C, Ghim J, Ryu SH. *Mechanisms regulating intestinal barrier integrity and its pathological implications*. Exp Mol Med. 2018 Aug 16;**50**(8):1-9. doi: 10.1038/s12276-018-0126-x.
4. Cereijido M, Contreras RG, Flores-Benítez D, Flores-Maldonado C, Larre I, Ruiz A, Shoshani L. *New diseases derived or associated with the tight junction*. Arch Med Res. 2007 Jul;**38**(5):465-78. doi: 10.1016/j.arcmed.2007.02.003.
5. Park JH, Kotani T, Konno T, Setiawan J, Kitamura Y, Imada S, Usui Y, Hatano N, Shinohara M, Saito Y, Murata Y, Matozaki T. *Promotion of Intestinal Epithelial Cell Turnover by Commensal Bacteria: Role of Short-Chain Fatty Acids*. PLoS One. 2016 May 27;**11**(5):e0156334. doi: 10.1371/journal.pone.0156334.

**Decellularized aortic homograft and pedicled m. latissimus dorsi flap: an innovative approach for tracheal reconstruction after button battery ingestion in infants.**
ID: 913

**Kategorie: DGKCH - Kinderchirurgische Forschung**

Joachim F. Kübler[1], Murat Avsar[1], Tobias Goecke[1], Oliver Keil[1], Harald Köditz[1], Nicolaus Schwerk[1], Alexander Horke[1], Gregor Warnecke[2], Benno Ure[1], Patrick Zardo[2]

[1]*Medizinische Hochschule Hannover, Hannover, Deutschland*, [2]*Universitätsklinikum Heidelberg, Heidelberg, Deutschland*

**Background:**
In recent years the incidence of button battery ingestions in infants and children has increased. Once lodged in the esophagus, batteries cause acute and delayed tissue damage. Complicated cases with tracheoesophageal fistula (TEF) require surgical treatment which is associated with a significant morbidity. We describe an innovative technique to manage these complex cases.

**Materials and methods:**
Retrospective and follow-up analysis of four patients aged < 18 months undergoing repair of a traumatic TEF in our institution in the period of 2018 - 2021. After initial stabilization, surgical repair was performed with extracorporeal membrane oxygenation (ECMO) support. The esophageal defect was resected and the tracheal defect bridged with a decellularized aortic homograft that was buttressed by a pedicled m. latissimus dorsi flap.

**Results:**
Tracheal reconstruction was performed uneventfully in all four patients. There were no intraoperative complications. Three patients required temporary cervical esophageal diversion and a secondary repair by gastric pull-up. In one patient immediate esophageal repair was feasible. All children survived with acceptable morbidity. No patient requires tracheostomy or ventilator support after a mean follow-up of 1.7 years.

**Conclusion:**
We describe for the first time the use of decellularized aortic homografts for tracheal reconstruction in infants. Our multidisciplinary approach and the use of bioprosthetic material in combination with a vascularized tissue flap was successful in managing complicated tracheoesophageal fistulas after battery ingestion in infants.

**The use of honey in button battery ingestions: A systematic literature review**
ID: 939

**Kategorie: DGKCH - Kinderchirurgische Forschung**

Yannick Michael Schmidt[1], Oliver Muensterer[1], Danielle Wendling-Keim[1]

[1]*LMU Klinikum, Dr. von Haunersches Kinderspital/Kinderchirurgie, München, Deutschland*
**The use of honey in button battery ingestions: A systematic literature review**

**Background:**
Button battery ingestions can cause severe and possibly fatal complications, especially if the battery is in the esophagus. The application of oral honey has recently been introduced by the National Capital Poison Center in the USA and in an ESPGHAN position paper in Europe, but clinical trials and experimental studies are limited. The goal of this systematic review was to analyze the evidence for this approach.

**Materials and methods:**
A systematic review of clinical trials and experimental studies analyzing the oral application of honey after button battery ingestion in children was performed. Inclusion criteria according to the PICO format were patient age 0-18 years, ingestion of BB, oral administration of honey or other substances, complication rate, esophageal injury, and mortality. A manual search in the databases was performed to identify relevant search terms to form the following queries to construct the extensive search. Search terms were the following: MEDLINE: ("Therapeutic Irrigation"[MeSH Terms] OR "Honey"[MeSH Terms] OR "Honey"[Text Word] OR "sirup"[Text Word] OR "glucos*"[Text Word] OR "sugar*"[Text Word] OR "therapeutic irrigat*"[Text Word]) AND ("electric power supplies/adverse effects"[MeSH Terms] OR "electric power supplies/methods"[MeSH Terms] OR "electric power supplies/trends"[MeSH Terms] OR "button batter*"[Text Word]), Web of Sciene: (honey OR sirup OR glucos* OR sugar* OR “therapeutic irrigate*”) AND “button batter*“), Cochrane Central Register of Controlled Trials (Central): ((MeSH descriptor: [Honey] this term only) OR (MeSH descriptor: [therapeutic irrigation] this term only) OR Honey OR sirup OR glucos* OR „therapeutic irrigation“ OR „therapeutic irrigations“) AND ((MeSH descriptor: [Electric power supplies] this term only) OR „button battery“ OR „button batteries“.
Furthermore, the search was extended by using snowballing on the reports reference lists. In Medline 26 results were retrieved, in Web of Science: 14 results und Cochrane 0 results. After reviewing the articles only 3 reports met our inclusion criteria.

**Results:**
We detected three publications that investigated the effects of honey after button battery ingestion. Two of these presented experimental in vitro and in vivo results and one a clinical retrospective study of 8 patients. There were two studies that reported in vitro studies with goat and piglet cadaver esophagi each. One study exposed the esophagus to the BB for 30 to 120 minutes, the other study for 30 minutes to 24 hours. Various substances were tested parallel to honey, and honey seemed to be more protective than the other substances. Nevertheless, in vitro conditions were different so that the two studies are not comparable and only 8 (Anfang et al., 2018) and 10 (Gyawali et al,2018) experiments, respectively, were performed with honey. The in vitro studies were followed by in vivo experiments by Anfang et al, 2018, with 9 piglets, here only 2 piglets were treated with honey, and they presented with less severe ulcers than the remaining 7 piglets treated with carafate or saline, however, exposure time was only 60 minutes. The only study in humans that we found included 8 patients in a retrospective study by Chandran et al., 2022, and only 2 patients were treated with honey, and, notably, these two patients did not suffer from any complications. Further outcome parameter except the severity of the ulcer and perforation were the pH and temperature, both of which were influenced by honey in a beneficial way.

**Conclusion:**

Follow up studies are required to further elucidate the effectiveness of the treatment with honey. The time intervals in which the use of honey is effective is not clear. Furthermore, a physiological model is needed for the in vitro testing possibly mimicking the peristalsis and the flow of the applied substances. However, current research points to honey as a widely available treatment option to apply during the waiting time to endoscopic removal of the BB.

**Nerve lesions in humeral shaft fractures in children**
ID: 495

**Kategorie: DGKCH - Komplikationen in der Kindertraumatologie**

Markus Dietzel[1], Aaron Flühr[1], Simon Scherer[1], Hans Joachim Kirschner[1], Jörg Fuchs[1], Justus Lieber[1]

[1]*Universitätsklinikum Tübingen Abteilung für Kinderchirurgie mit Poliklinik, Tübingen, Deutschland*

**Background:**
Humeral shaft fractures in childhood and adolescence are rare, compared to fractures of the proximal humerus, and supracondylar as well as condylar injuries. Depending on fracture type and displacement, treatment is conservative using immobilization, or surgical usually by elastic nailing (ESIN). Lesions of the large arm nerves may occur both accidentally due to the initial trauma and iatrogenically during surgical treatment. The purpose of this study is to evaluate the incidence and nature of nerve lesions in humeral shaft fractures in children and adolescents, and to provide diagnostic and therapeutic options as well as outcome information.

**Materials and methods:**
Evaluation of all humeral shaft fractures in children and adolescents from birth to completed 16 years of age in a ten-year period at a pediatric traumatology center was performed. Analysis of epidemiology and care with focus on nerve lesions was carried out. Causes, diagnosis and treatment, as well as outcome of cases with lesions of the large arm nerves were evaluated. A positive vote of the responsible ethics committee is available.

**Results:**
During the evaluation period from 08/2010 to 12/2020, 42 children with humeral shaft fractures were treated. The average age was 7 years with a balanced gender ratio. The predominant accident cause was a trivial fall in 69% of cases, with sports and traffic accidents and child abuse in the remaining cases. Transverse fractures were leading with 55%, followed by oblique (31%), spiral (9%), and multiple fragment fractures (5%). Thirty of 42 children and adolescents (71%) were hospitalized with a mean length of stay of 3.65 days (1–8 days). 24 children and adolescents (57%) underwent osteosynthesis with ESIN as the leading method in 18 cases (43%). A deficit in the radial nerve area (sensory and/or motor) was noted postoperatively in 6 cases (14%). This had been documented preoperatively as tingling paresthesia in only one case, and was evident postoperatively in the other cases. All cases with nerve lesion occurred in fractures with osteosynthesis (ESIN n=4, in one case secondary after external fixator, as well as plate and screw osteosynthesis, each n=1). Neurosurgical evaluation (n=4) including nerve sonography and neurophysiological examination led to surgical interventions in cases of pronounced nerve damage with short-term open revision with neurolysis two weeks after ESIN (n=1) and open neurolysis in the context of planned metal removal (plate) after six months (n=1). Restitutio ad integrum was documented in all cases.

**Conclusion:**
Diaphyseal humeral fractures in childhood are rare and represent an entity with special challenges. On one hand, this applies to osteosynthesis in this area, for which plates and screws are available in addition to ESIN. On the other hand, due to the anatomical proximity especially for the radial nerve, there is an increased risk of injury here, both from the initial trauma and iatrogenically. This requires a well-considered and surgically experienced approach in order to achieve the goal of an exact anatomical reduction with stable osteosynthesis without further lesions. If nerve injuries occur, interdisciplinary clarification and, if necessary, early intervention are mandatory, in order not to diminish the overall good prognosis.

**The Role of Surgery in the Treatment of Metastases with rare Localizations in Neuroblastoma**
ID: 268

**Kategorie: DGKCH - Onkologie in der Kinderchirurgie**

Simon Scherer[1], Jörg Fuchs[1], Michael Esser[2], Peter Lang[3], Andreas Schmidt[1]

[1]*Universitätsklinikum Tübingen Abteilung für Kinderchirurgie mit Poliklinik, Tübingen, Deutschland*, [2]*Radiologische Universitätsklinik Tübingen, Tübingen, Deutschland*, [3]*Universitätsklinikum Tübingen - Klinik für Kinderheilkunde und Jugendmedizin, Tübingen, Deutschland*

**Background:**
As about half of the children with neuroblastoma show metastasis it is a frequent issue for pediatric oncology surgeons, whether metastatic surgery should be performed, so the present work tries to sum up the current therapies for neuroblastoma metastases, presents three cases with rare localizations or tumors that were refractory to therapy, and assesses conclusions of the authors' experiences.

**Materials and methods:**
The department of Pediatric Surgery and Pediatric Urology of the University Children's Hospital in Tuebingen, Germany is a national surgical reference center for various solid tumor entities, including neuroblastoma. All children presented in this publication were operated on by the chief physician, Prof. Dr. med. Jörg Fuchs. We retrospectively analyzed the data from the last ten years of patients who underwent surgery for neuroblastoma in our department.

**Results:**
Three cases of rare metastases in pediatric Neuroblastoma patients are reported. One patient had a metastasis in the glans penis. This localization is described here for the first time in a neuroblastoma patient. Another patient had two bone-marrow-transplantations with irradiation of a metastasis in the proximal ulna and immunotherapy with dinutuximab and performed resection on this vital metastasis. The third patient was 6.5 years old and had a metastasis in the pancreatic body. Since no definite statements regarding metastatic surgery can be made for neuroblastoma to date, we conducted current research and evaluated the role of the surgeon.

**Conclusion:**
Although children with neuroblastoma often present with metastases, there are no clear (and no uniform) recommendations (between the different international study groups) regarding the treatment of metastases which after standard therapy persist, increase or are still vital. Often, the metastases are irradiated with potential radiation damage. However, surgery is also indicated in selected cases and for rare localizations.

**Abdominal compartment syndrome and its treatment in neuroblastoma stage 4S with severe hepatomegaly.**

ID: 533

**Kategorie: DGKCH - Onkologie in der Kinderchirurgie**

Till-Martin Theilen[1], Michael Merker[2], Konrad Bochennek[3], Henning Cornelius Fiegel[1], Udo Rolle[1]

[1]*University Hospital Frankfurt, Department of Pediatric Surgery and Pediatric Urology, Frankfurt am Main, Deutschland*, [2]*University Hospital Frankfurt, Department of Pediatric Stem Cell Transplantation and Immunology, Frankfurt am Main, Deutschland*, [3]*University Hospital Frankfurt, Department of Pediatric Hematology and Oncology, Frankfurt am Main, Deutschland*

**Background:**

Abdominal compartment syndrome (ACS) due to gross tumor volume is a rare complication in neuroblastoma stage 4S. The aim of this case series is to report the individual course of three patients with neuroblastoma stage 4S, who required prolonged laparostomy for ACS release.

**Materials and methods:**

A retrospective review of the medical and operative charts was performed of three patients diagnosed with neuroblastoma stage 4S and ACS at our institution between 2017 and 2019.

**Results:**

All three patients diagnosed with a neuroblastoma stage 4S developed severe ACS. Massive tumor volume of the primary tumor and extensive hepatomegaly due to liver metastasis caused a pressure related abdominal organ failure. Emergency laparostomy led to the recovery of kidney and lung function in all three patients. The patients age at laparostomy was 3, 5, and 6 months. A silicon patch closure of the abdominal wall combined with a vacuum dressing was applied and changed weekly. Abdominal wall closure was achieved after 138 days in two patients. Both patients survived. One patient died on day 21 due to progression of disease.

**Conclusion:**

ACS is a rare but life-threatening complication in neuroblastoma stage 4S. Early extensive laparostomy using an abdominal wall patch and vacuum therapy combined with chemotherapy and intensive care lead to the survival of 2 out of 3 affected patients. This treatment algorithm requires a true multidisciplinary approach by pediatric oncology, pediatric intensive care and pediatric surgery. We advocate an early, aggressive surgical treatment, i.e. laparostomy combined with patch and vacuum-therapy, in these patients.

### Congenital lung malformations –The Hamburg experience a retrospective clinical study

ID: 602

**Kategorie: DGKCH - Onkologie in der Kinderchirurgie**

Melanie Le[1], Christian Tomuschat[1], Konrad Reinshagen[1]

[1]*Department of Pediatric Surgery, University Medical Center Hamburg-Eppendorf, Hamburg, Hamburg, Deutschland*

**Background:**

Congenital pulmonary airway malformations (CPAM), pulmonary sequestration (BPS), congenital lobar emphysema (CLE), and bronchogenic cysts are the most prevalent congenital lung malformations. An increased risk of bronchopulmonary infections is a feature shared by all pulmonary abnormalities. We would like to provide our case numbers and our experiences with a particular focus on the therapy decision and surgical technique over the last seven years in this single-center retrospective analysis. Given the rarity of these diseases, thorough examination of the gathered data is crucial and advantageous for the field of pediatric surgery. Here, we provide our initial work on CPAMs.

**Materials and methods:**

A retrospective, single-center clinical investigation was carried out on patients treated for congenital lung abnormalities at the University Medical Center Hamburg-Eppendorf (UKE). Selected ICD codes were used to identify cases (Q33.0; Q33.2; QQ33.4, 33.6; Q33.8; Q33.9). The data were collected between 2015 and 2022. Patients were included up to the age of 18. Demographic information was obtained from the electronic case record, including age at diagnosis, clinical presentation, surgical technique, time from incision to suture, intraoperative and postoperative problems, length of hospital stay, and subsequent clinical course.

**Results:**

Over seven years, 40 cases were identified; of these, 27 were CPAM, seven were BPS, four were bronchogenic cysts, and two were CLE. 70.4% of CPAM were operated and 22.2% were conservatively treated with observation only. On average, 2.1 CPAM surgeries are performed per year in our center. 11/19 of the operated CPAM were symptomatic. CPAM presented most frequently with mediastinal shift (35.7%), in 28.6% of cases with postnatal adjustment disorder then with recurrent infections (21.4%). The average age at surgery was 5.33 months. No relevant intraoperative or postoperative complications occurred with either surgical technique (open, thoracoscopic). The thoracoscopic procedure resulted in a mean operative time of 88 minutes vs. 138 minutes for open approach. The length of stay was an average of 6 days vs. 17 days (thoracoscopic vs. open). Survival after 1 and if given 5 years was achieved by all patients, regardless of the treatment regimen or the chosen surgical procedure. One case of the operated children (1/27) continued to show recurrent infections postoperatively (>2 infections/year).
Thoracoscopic surgery is the recommended treatment for symptomatic congenital lung lesion. However, surgical complications for asymptomatic lesions must be balanced against the risks of recurring infections and potential malignant transformation. With a mean age of 6.03 months at surgery, asymptomatic children in our group tended to be older than symptomatic patients, whose mean ages were 4.42 months. The fact that no recurring infections were found in any of the cases that were treated conservatively suggests that small asymptomatic lesions can be observed. However, the prevalence of malignant transformation has yet to be determined and is an ongoing concern, often leading to the decision to operate on these patients. Generally, re-evaluation is advised at the age of 6 months and in a symptom-free-state. The surgical approach resulted in a generally low intraoperative and postoperative complication rate. The most frequent postoperative consequence was a low-grade pneumothorax with self-limiting further course. Since it was unable to reliably distinguish healthy tissue from pathological tissue in two cases, an open thoracotomy after initial thoracoscopy had to be performed. Our data demonstrate that the thoracoscopic method results in lower operating times and hospital stays (88 minutes vs. 138 minutes; 6 days vs. 17 days). Although the thoracoscopic surgery seems advantageous, it is not always feasible and must be considered against the patients' safety. Due to the rarity of this condition, there are only 2.1 CPAM surgeries performed each year. The number of operations for CPAMS per year stayed the same over the course of the records; there was no increase in operations brought on by improved prenatal ultrasonography diagnosis.

Further analysis for BPS, CLE and bronchogenic cysts are currently in progress.

**Conclusion:**
The need for surgical resection in each case must be determined on an individual basis. It is feasible to wait until the sixth month of life for a CT of the thorax to re-evaluate a surgical rationale in the case of symptom-free, minor symptoms and/or smaller CPAM finding. Intraoperative and postoperative problems are rare with surgical treatment. The need for a thoracoscopic surgery is apparent. Surgery for congenital lung malformations continues to be uncommon overall. Our CPAM analysis included 19 surgeries (n=27), hence larger research with higher case number is required to reliably predict the outcome.

**Chromosome 8 amplification drives poor patient outcome via expression of 4E-BP1 in Ewing sarcoma**
ID: 769

**Kategorie: DGKCH - Onkologie in der Kinderchirurgie**

Cornelius Funk[1], Martin Orth[1], Karim Aljakouch[2], Anna Ehlers[1], Jing LI[1], Tilman Hölting[1], Rainer Will[3], Franziska Willis[4], Endrit Vinca[1], Shunya Ohmura[1], Roland Imle[5,6], Maximilian Knott[7], Felina Zahnow[1], Francisco Javier Alonso[8], Felix Sahm[9,10], Martin Schneider[4], Ana Banito[5], Gabriel Leprivier[11], Wolfgang Hartmann[12], Uta Dirksen[13], Olaf Witt[14], Ina Oehme[15], Stefan Pfister[16], Laura Romero-Perez[1], Jeroen Krijgsveld[2], Florencia Cidre-Aranaz[1], Thomas Grünewald[1,17], Julian Musa[1,4]

[1]*Division of Translational Pediatric Sarcoma Research (B410), German Cancer Research Center (DKFZ), Heidelberg, Deutschland*, [2]*Division of Proteomics of Stem Cells and Cancer, German Cancer Research Center (DKFZ), Heidelberg, Deutschland*, [3]*Cellular Tools Core Facility, German Cancer Research Center (DKFZ), Heidelberg, Deutschland*, [4]*Department of General, Visceral and Transplantation Surgery, University Hospital Heidelberg, Heidelberg, Deutschland*, [5]*Soft-Tissue Sarcoma Junior Research Group, German Cancer Research Center (DKFZ), Heidelberg, Deutschland*, [6]*Division of Pediatric Surgery, University Hospital Heidelberg, Heidelberg, Deutschland*, [7]*Department of Neurobiology, Faculty of Life Sciences & Sagol School of Neuroscience, Tel Aviv, Israel*, [8]*Pediatric Solid Tumour Laboratory, Institute of Rare Diseases Research (IIER), Instituto de Salud Carlos III, Madrid, Spain*, [9]*Clinical Cooperation Unit Neuropathology (B300), German Cancer Research Center (DKFZ), Heidelberg, Deutschland*, [10]*Department of Neuropathology, University Hospital Heidelberg, Heidelberg, Deutschland*, [11]*Institute of Neuropathology, University Hospital Düsseldorf, Düsseldorf, Deutschland*, [12]*Division of Translational Pathology, Gerhard-Domagk-Institute of Pathology, University Hospital of Münster, Münster, Deutschland*, [13]*Department of Pediatric Hematology and Oncology, University Hospital of Essen, Essen, Deutschland*, [14]*Department of Pediatric Oncology, Hematology, Immunology and Pulmonology, Heidelberg University Hospital, Heidelberg, Deutschland*, [15]*Clinical Cooperation Unit Pediatric Oncology, German Cancer Research Center (DKFZ), Heidelberg, Deutschland*, [16]*Division of Pediatric Neurooncology, German Cancer Research Center (DKFZ), Heidelberg, Deutschland*, [17]*Institute of Pathology, University Hospital Heidelberg, Heidelberg, Deutschland*

**Background:**
To refine patient care in the context of precision oncology, it is increasingly important to understand mechanisms underlying inter-individual tumor heterogeneity, especially in oligomutated cancers, to refine patient care. Ewing sarcoma (EwS) are genetically characterized by pathognomonic *FET::ETS* gene fusions (in most cases *EWSR1::FLI1*) and exhibit a paucity of other recurrent somatic alterations that might account for observed broad variations in clinical patient outcome. Following *FET::ETS* fusions, chromosome (chr) 8 amplification is the second most common recurrent somatic alteration in

EwS. However, the pathophysiological role and potential clinical implication of chr8 amplification in EwS remain unclear.

**Materials and methods:**
We performed integrative analyses of microarray and RNA sequencing gene expression data as well as methylome profiling data from EwS tumor samples with corresponding clinical patient data. Furthermore, functional RNA interference experiments *in vitro* and *in vivo* as well as multi-omics profiling (including transcriptomic profiling by gene expression microarrays and proteomic profiling using masspectrometry) of EwS cell lines with and without targeted RNA interference was performed.

**Results:**
Here, we report that chr8 amplification gene expression signatures are significantly associated with poor overall survival of EwS patients, and that this effect is predominantly mediated by expression of the translation initiation factor binding protein EIF4EBP1 (4E-BP1). High *EIF4EBP1* expression shows the strongest association with poor overall survival of EwS patients among all genes located on chr8, which as well holds true in a subgroup analysis of patients only harboring localized disease, emphasizing the prognostic role of 4E-BP1 in EwS. Consistently, chr8 amplification was associated with higher intra-tumoral *EIF4EBP1* expression in EwS patient samples. RNA interference-mediated knockdown of 4E-BP1 significantly decreased proliferation, clonogenicity, and spheroidal growth of EwS cells *in vitro* as well as tumor growth of EwS xenografts *in vivo*. To identify potential mechanisms via which EIF4EBP1 mediates its phenotypic effect, we performed integrated proteomic and transcriptomic profiling of EwS cell lines with and without silencing of 4E-BP1 revealing that 4E-BP1 guides a multifunctional proteomic network including hubs associated with RNA processing, translational regulation, and chromatin modification.

**Conclusion:**
Collectively, we establish a significant association between chr8 amplification gene expression signatures and poor overall survival in EwS. We show that this association primarily depends on the expression of the translation initiation factor binding protein 4E-BP1 regulating a multifunctional proteomic network. Our data suggest that cytogenetic testing for chr8 amplifications in EwS patient tumor samples may help to improve risk-stratification and therapy decision making in the context of precision oncology.

**Catheter Implanation in children unter peritoneal dialysis: 20 years of expertise.**
ID: 563

**Kategorie: DGKCH - Unklare Raumforderung des Abdomens: Diagnostik und Therapie**

Michail Aftzoglou[1], Johanna Hagens[1], Nariman Mokhaberi[1], Gerhard Schön[2], Jun Oh[3], Konrad Reinshagen[1], Christian Tomuschat[1]

[1]*University Medical Center Hamburg Eppendorf, Department of Pediatric Surgery, Hamburg, Deutschland*, [2]*University Medical Center Hamburg Eppendorf, Institute of Medical Biometry and Epidemiology, Hamburg, Deutschland*, [3]*University Medical Center Hamburg Eppendorf, Department of Pediatrics, Hamburg, Deutschland*

**Background:**
A Tenckhoff catheter implant is typically indicated by insufficient renal filtration and urine output. However, examining the underlying disease in conjunction with the other underpinning characteristics of these children can identify common features that set them up for difficulties. As a result, the results of this study, will help to identify frequent, which will improve peritoneal dialysis.The purpose of this study is to examine the medical files of patients who underwent Tenckhoff Catheter implantation at our clinic, in order  to ascertain the risks and limitations of peritoneal dialysis in children. To identify the risk factors for anticipated problems we aim to generate a risk score.

**Materials and methods:**
File Records of Children undergone a Tenckhoff Implantations were collected using the operative code from our facility's operations database system. A total of 302 Implantations operations were retrieved between 2002 and 2022. Following an assessment of the surgical records, 243 patients were identified. Distribution of ages, the presence of primary and secondary diseases, the length of peritoneal dialysis and its side effects, overall survival, and the requirement for kidney transplantation were noted. Cases of catheter infection or malfunction received particular attention. The prevalence of hernias and various additional surgeries were noted. An examination of time to event and Cox regression completed the analysis. The Clavien-Dindo Classification was used to classify complications.

**Results:**
34,2% of the 302 implants performed on 243 individuals were in newborns or infants, while 32,5% were in toddlers. The incidence was the same for both genders. 66,7% of the population had primary renal disease, and 74 of them underwent kidney transplantation. The most frequent complications included peritonitis and catheter malfunction brought on by dislocation or occlusion. 41 patients, or 24.4% more than once, required reimplantation. 17,2% of implant surgeries since 2013 have included omentum resection. 27,8% of the implantations had peritonitis recorded, and S. epidermidis or E. coli were the most common specimens isolated. 37 patients underwent a herniotomy after catheter implantation.

**Conclusion:**

Although Tenckhoff implantation is still a minimally invasive procedure, patient and treatment variables are crucial for peritoneal dialysis effectiveness. Therefore, understanding typical catheter usage pitfalls as well as risk factors in the patient's past history can enhance the effectiveness of the treatment and the quality of the patient's life.

**IFTT in children and adolescents - A case series and systematic review of literature**
ID: 628

**Kategorie: DGKCH - Unklare Raumforderung des Abdomens: Diagnostik und Therapie**

Johannes Meyer[1], Frank-Mattias Schäfer[1], Maximilian Stehr[1]

[1]*Diakoneo Cnopfsche Kinderklinik, Abteilung für Kinderchirurgie und Kinderurologie, Nürnberg, Deutschland*

**Background:**
Isolated fallopian tube torsion (IFTT) is defined by rotation of the fallopian tube around itself without involving the ipsilateral ovary. It is a rare cause of acute lower abdominal pain in (adolescent) girls but is commonly overlooked. In sonographic examinations it can appear as indistinct mass in the lower abdomen. Due to its rarity, literature is still scarce in this age group. Currently, there is no generally accepted management and treatment. Herein we report a case series of three girls along with a systematic review of literature.

**Materials and methods:**
To identify and review cases of IFTT we retrospectively searched all cases treated at our department in the last fifteen years. Additionally, a systematic literature research on pediatric IFTT was carried out on Medline/ Pubmed database according to PRISMA principles using predefined search terms and inclusion criteria. Patient characteristics regarding age, clinical symptoms, diagnostic methods, treatment, concomitant pathologies and follow-up were analyzed.

**Results:**
Three patients were identified in our institution, Additionally, 60 articles from 1981 to 2021 with 167 patients were included. Overall, 170 girls with diagnosed IFTT were included in the analysis. Mean age was 13.0 years. Left tube was more commonly affected (52.9%). Abdominal pain was present in 99.4% of cases accompanied with nausea in 57.1%. Sonographic evaluation was the most commonly used diagnostic tool (93.5%) followed by CT study (25.9%). In 16.4% correct preoperative diagnosis was made. Laparoscopy was performed in 80.2% versus conventional surgery in 19.7%. Salpingectomy was the most common treatment option in 111 cases (66.9%) and 55 (33.1%) patients were treated with detorsion of the tube (organ-sparing management). Girls with symptoms longer than one day had significant higher rate of salpingectomy (95% CI, *P*= 0,0323).

**Conclusion:**
Overall, data quality regarding IFTT in children and adolescents is still poor. When IFTT is suspected, diagnostic emergency laparoscopy should be performed to possibly preserve future reproductive potential. In case of detorsion and reinstated blood supply organ-preserving management should be performed with simultaneous addressment of concomitant pathology if possible.

**Spillage Preventing Surgery for Giant Ovarian Cysts in Children and Adolescents: A Case Series**
ID: 630

**Kategorie: DGKCH - Unklare Raumforderung des Abdomens: Diagnostik und Therapie**

Illya Martynov[1,1], Maria Mangova[2], Manuela Siekmeyer[2], Robin Wachowiak[1], Franz Wolfgang Hirsch[3], Martin Lacher[1]

[1]*University of Leipzig, Department of Pediatric Surgery, Leipzig, Deutschland*, [2]*University of Leipzig, Department of Pediatrics, Leipzig, Deutschland*, [3]*University of Leipzig, Department of Pediatric Radiology, Leipzig, Deutschland*

**Background:**
The surgical management of giant ovarian cysts (≥10 cm in diameter) is challenging. Avoiding contamination of the peritoneal cavity by the leakage of cyst fluid is mandatory. The aim of this study was to evaluate the safety, effectiveness, and feasibility of spillage preventing surgery for giant ovarian cysts.

**Materials and methods:**
After Pfannenstiel incision a wound retractor (Alexis™, Size XS) was placed directly through the fascia. The cyst wall was glued to a 3M™ Steri Drape using 2-Octyl-Cyanoacrylat (Dermabond™). After placement of a purse-string suture on the cyst wall a pleural drainage needle catheter was inserted into the cyst and the cyst fluid was aspirated. The puncture site was closed, the cyst was exteriorized and an ovarian cystectomy was performed.

**Results:**
Seven patients were included in the series. The mean age at operation was 15.3 ± 1.6 years and mean BMI was 24.1 ± 6.0. Symptoms at presentation varied, including abdominal pain, nausea, vomiting, and weight gain. One patient was asymptomatic. All patients presented with a normal CA 125, LDH, ß-HCG, and AFP. The diagnosis was made by ultrasonography and MRI. Median volume of the cysts was 3.9 liter (range, 0.3 – 31.9). Four patients underwent ovarian cystectomy, two salpingectomy, and one salpingo-oophorectomy. Mean operating time was 223.2 ± 112.8 min. No intraoperative or postoperative complications occurred. Histopathology confirmed five tumors as cystadenomas (unilocular serous, n=3; mucinous, n=2) and two as mature cystic teratomas.

**Conclusion:**
In our small case series the spillage preventing technique was safe and feasible with favorable cosmetic results.

**Vesicoureteral Reflux in Duplex Kidneys – A 20 years institutional experience**

ID: 253

**Kategorie: DGKCH - VUR**

Benedikt Richard Wagner[1], Ioan Cristian Urla[1], Verena Ellerkamp[1], Tobias Luithle[1], Jürgen Schäfer[2], Jörg Fuchs[1]

[1]*Universitätsklinikum Tübingen Abteilung für Kinderchirurgie mit Poliklinik, Tübingen, Deutschland*, [2]*Radiologische Universitätsklinik Tübingen, Kinderradiologie, Tübingen, Deutschland*

**Background:**

The clinical presentation and treatment of vesicoureteral reflux (VUR) in duplex kidneys (DK) differs widely due to anatomic and clinical variability of the duplicated renal system. The aim of our study was to analyze the treatment outcomes of VUR in relation to the different anatomical conditions and to analyze the therapy in relation to the development of renal function in children.

**Patients and methods:**

Between March 2002 and September 2022, 148 patients (91 female, 57 male) with duplex kidney were treated at our institution for VUR. The diagnostic work-up consisted of voiding cystography, renal ultrasound and renal function testing (magnetic resonance urography or renal scan). A retrospective analysis of patient charts was carried out.

**Results:**

The median age at surgery was 17 months (range 0 - 168). Sex ratio was female: male = 3: 2. The vast majority of the patients (n=118, 80%) presented with urinary tract infection. In 19 cases (13 %) the condition was prenatally diagnosed. Less common symptoms included flank pain (n=7, 5%) and enuresis (n=4, 3%). 126 patients (85%) had unilateral duplex kidneys (51 right, 74 left). Bilateral duplex collecting system was present in 22 patients (15%). Complete ureteric duplication was observed in 118 patients (80%), while 30 patients presented an incomplete duplication (20%). The grade of VUR was moderate in the majority of the cases (VUR Grade III-IV, 89 patients, 60%), low-grade VUR was encountered in 43 patients (29 %). Only 16 patients (11%) presented with high-grade VUR. The initial surgical treatment consisted of: subureteral injection (SU) in 57 patients (38%), ureterocystoneostomy (UCN) in 54 (37%) and heminephroureterectomy (HNU) in 37 (25%). After a median follow-up of 34 months (1 – 173), 104 patients were free of symptoms, while 30 patients required additional surgical intervention: 18 patients (10 Re-SU, 7 UCN, 1 HNU) after SU, 10 patients (3 Re-UCN, 3 SU, 4 HNU) after UCN and 2 patients (1 SU and 1 resection of ureteral stump) after HNU. Preservation of renal function could be achieved in all patients.

**Conclusion:**

The surgical approach for the treatment of VUR in children with duplex kidney should be determined on an individual basis, as it is dependent on anatomical condition and existing clinical symptoms. Regardless of the surgical therapy, preservation of differential renal function can be achieved.

**Vesicoscopic ureterocystoneostomy for vesicoureteral reflux and primary obstructive megaureter in children: a retrospective single-centre study**

ID: 289

**Kategorie: DGKCH - VUR**

Alexandra Wilke[1], Christian Kruppa[1], Katrin Schuchardt[1], Thomas Kosk[1], Guido Fitze[1]

[1]*Universitätsklinikum Dresden, Klinik für Kinderchirurgie, Dresden, Deutschland*

**Background:**

What is the advantage of minimally invasive vesicoscopic ureteral reimplantation (v-UCN) in the treatment of vesicoureteral reflux or primary obstructive megaureter in childhood? Which surgical techniques are feasible? We compared endoscopic with open surgical procedures in terms of operating time, postoperative course and safety.

**Materials and methods:**

Ureterocystostomy (UCN) was performed in 260 patients. A total of 155 were treated vesicoscopically according to Cohen or Leadbetter-Politano procedures. The control group consists of patients who received open ureteral reimplantation or Lich-Gregoir-antirefluxplasty. This retrospective study analyses children who underwent surgery between February 2010 and June 2022 and were diagnosed with vesicoureteral reflux (VUR) or primary obstructive megaureter (POM).

**Results:**

All v-UCN started could be performed safely. The vesicoscopically operated patients left the hospital significantly earlier and had a lower need for pain medication than children in the control group. Similarly, the need for bladder decompression by bladder catheter or cystofix was shorter in the minimally invasive patients. Overall, there was no significant difference in the incidence of VUR-recurrences when comparing the open and vesicoscopic methods. The vesicoscopic procedure took longer than the same technique performed with open surgery.

**Conclusion:**

Vesicoscopic UCN is a safe form of therapy when indicated for VUR or POM in children. Reduced need for pain medication, shorter bladder drainage, faster mobilisation and thus shorter hospital stay show that this method offers advantages to patients. The longer operation times are within the tolerable range. The vesicoscopic technique offers the opportunity for further development of minimally invasive reconstructive skills including structured assistant training in a safe surgical environment.

**Resolution of infantile congenital hydronephrosis – experiences of a tertiary referral hospital**
ID: 543

**Kategorie: DGKCH - VUR**

Sabine Riessle[1], Marcus Weitz[2], Tobias Luithle[3], Jörg Fuchs[3], Verena Ellerkamp[3]

[1]*Eberhard Karls Univsersität, Medizinische Fakultät, Tübingen, Deutschland*, [2]*Universitätsklinikum Tübingen, Kindernephrologie, Tübingen, Deutschland*, [3]*Universitätsklinikum Tübingen, Kinderchirurgie und Kinderurologie, Tübingen, Deutschland*

**Background:**
Congenital hydronephrosis (CH) accounts for the majority of congenital anomalies of the kidney and urinary tract (CAKUT). The resolution rate of Society for Fetal Urology (SFU) grade 1–2 hydronephrosis is reported to be as high as 98%. Ureteropelvic junction obstruction (UPJO) is reported to be the most frequent underlying condition of CH, accounting for 35–60% of all cases, followed by vesicoureteral reflux (VUR) and ureterovesical junction obstruction (UVJ). The aim was to evaluate these rates in our own patient cohort in order to possibly modify our standardized follow-up program in these patients.

**Materials and methods:**
All data sheets of patients referred with CH in 2018 were analysed retrospectively concerning the following: age, sex, SFU grade at first and last appointment, urinary tract infections (UTI), underlying condition (UPJO, VUR, UVJ), loss of function. Endpoints were surgical intervention or maturation.

**Results:**
In 2018 79 children (54 male) presented with CH in 110 renal units (RU) with a median age of 1 month (range 0-18; prenatal diagnosis n=36), predominantly on the left side (n=41, 39%). SFU grades were: grade 1 in 29 (26.4%), grade 2 in 30 (27,3%), grade 3 in 26 (23,6%), and grade 4 in 25 (22,7%) RU. The median follow-up was 25 months (range 0-50), 41 children (42.3%) developed urinary tract infections (UTI) over time. Micturating cystourethrogram (MCUG), renal scan, and magnetic resonance urography (MRU) was performed in 50, 62, and 21 patients, respectively. Underlying conditions of CH were UPJO, VUR, or UVJ in 16.4%, 36.4%, or 12.7% of all RU; in cases of mild CH (SFU grade 1,2), the rates were 8.5%, 33,9% or 5.1%. Surgery was performed in 39 patients; 39% of RU with mild CH, 55% of RU with SFU grade 3 or 4. Median age at surgery was 10 months (range 0-36) with a median observation period between initial presentation and surgery of 6 months (range 0-34). Operative procedures were cystoscopic subureteral injection in 30 (27.3%), ureteroneocystostomy in 13 (11.9%), and dismembered pyeloplasty in 12 (10.9%) RU. Resolution of CH was seen in 16 patients (32% of RU with mild CH, 10% of RU with SFU grade 3 or 4), 23 patients are still under control, one patient is lost to follow-up.

**Conclusion:**

There are relevant numbers of patients with mild CH with no resolution during follow-up as well as relevant numbers with the necessity for surgery over time. The most frequent underlying condition of CH in our patients was VUR. Standardized follow-up regimen for all infants with CH are mandatory.

**Robot-assisted laparoscopic ureteral reimplantation (RALUR) for vesicoureteral reflux (VUR): Preliminary Experience**

ID: 654

**Kategorie: DGKCH - VUR**

Martin Lacher[1], Gabriel Goetz[1], Illya Martynov[1], Oliver Johannes Deffaa[1], Jan-Hendrik Gosemann[1]

[1]*Klinik- und Poliklinik für Kinderchirurgie, Universitätsklinikum Leipzig, Leipzig, Deutschland*

**Background:**

Excluding endoscopic management with subureteral injection of bulking agents to date, the gold standard surgical procedure for primary (and secondary) VUR is still open ureteral reimplantation. In recent years robot-assisted laparoscopic extravesical ureteral reimplantation (RALUR) is being adopted at large pediatric urology centers worldwide. We report our preliminary experience with RALUR.

**Materials and methods:**

All RALUR were performed using the Da Vinci Xi system (Intuitive Surgical Sunnydale, CA). After introduction of a Foley catheter an 8mm camera port was placed at the level of the umbilicus, two 8mm working ports were introduced in the right and left abdomen and a 5mm assistant port was placed in the contralateral lower quadrant. A classic Lich-Gregoir RALUR was carried out.

**Results:**

From 11/2021 to 8/2022 seven patients with primary vesicoureteral reflux (VUR) underwent unilateral RALUR (two boys, five girls, median age 2.2 years (range 1.3-8.6); median weight 13.5 kg (range 9.0-39.2)). Three patients had grade 5 VUR, three grade 4 VUR and one patient grade 2 VUR. Three patients had anatomic anomalies: Duplex kidney (N=3), bifid ureter (N=1) and duplex ureter (N=2). The right side was affected in six cases, the left side in one child. Three patients underwent DexellTM injection prior to surgery, one patient had contralateral open antireflux procedure according to Politano-Leadbetter. After RALUR a cystoscopy with DexellTM injection on the contralateral side was performed in four patients with bilateral VUR. Mean operating time was 149±16 min in patients with additional cystoscopy and 114±11min with unilateral RALUR only. No intraoperative complications occurred. In one patient a 1mm perforation of the bladder mucosa was closed with a figure-8-stich. One girl was readmitted six days after surgery due to urinary retention which was self-limiting. Three patients were discharged on the 3rd postoperative day and four patients on the 4th postoperative day.

**Conclusion:**

Our limited experience suggests that RALUR is a viable minimally invasive surgical option for children with VUR with an excellent feasibility and steep learning curve. Short-term outcomes are acceptable. Due to the short follow-up long-term results need to be carefully assessed.
